# Intracortical Microstimulation in Brain–Computer Interfaces: Evoking Perception and Plasticity

**DOI:** 10.34133/cbsystems.0690

**Published:** 2026-09-11

**Authors:** Pengfei Hu, Chong Chen, Yunliang Zang, Xiaohong Li, Dong Ming

**Affiliations:** ^1^ Academy of Medical Engineering and Translational Medicine, Tianjin University, Tianjin 300072, China.; ^2^ Haihe Laboratory of Brain-Computer Interaction and Human-Machine Integration, Tianjin 300392, China.; ^3^ State Key Laboratory of Advanced Medical Materials and Devices, Tianjin 300072, China.; ^4^ Integrative Medical Center, Tianjin University, Tianjin 300162, China.

## Abstract

Intracortical microstimulation (ICMS), the activation of specific neuronal populations via microelectrodes implanted in the cortex, has emerged as a key technology for invasive brain–computer interfaces (BCIs). This article reviews the technical foundations and functional applications of ICMS within the BCI field, highlighting its diverse capabilities in both perception construction and targeted neuromodulation, while emphasizing the interface and parameter constraints that shape its long-term use. At the technical level, we analyze the evolution of ICMS microelectrode interfaces from rigid arrays to flexible, biomimetic, and biohybrid strategies, alongside key pulse-train parameters relevant to neural recruitment and application safety. Functionally, we discuss how biomimetic and spatiotemporally patterned ICMS generates high-resolution artificial tactile and visual perception, and how ICMS can serve as learnable information channels to guide behavior. We further consider temporally contingent and closed-loop ICMS as plasticity-based approaches for modulating cortical functional connectivity and pathological network activity in selected experimental models, while noting translational challenges related to stability, scalability, safety, and patient variability. Finally, we extend the discussion to novel biohybrid neural interfaces, including cell-seeded interface modifications, axon-guidance strategies, and stem-cell- and brain-organoid-integrated platforms, which provide a theoretical reference for next-generation biointegrated neuromodulation technologies in BCIs. Together, this review evaluates ICMS in BCIs along 2 central lines: evoking artificial perception and engaging plasticity-based modulation, while highlighting that long-term translation will require coordinated advances in interface reliability, stimulation encoding, closed-loop calibration, safety evaluation, and biohybrid integration.

## Introduction

Since the turn of the 21st century, brain–computer interfaces (BCIs) have continuously evolved, offering new hope for functional recovery in patients with neurological injuries, developmental disorders, and related conditions [1]. Neuromodulation technologies artificially activate the nervous system to inject information or modulate local cortical activity and serve as a fundamental technical pillar of BCIs [2,3]. Current neuromodulation technologies are broadly classified into noninvasive techniques, such as transcranial magnetic stimulation (TMS) [4] and transcranial direct current stimulation (tDCS) [5], and invasive techniques, such as epidural electrical stimulation [6], cortical electrical stimulation [7], intracortical microstimulation (ICMS) [8], and deep brain stimulation (DBS) [9]. Among these, ICMS has emerged as a prominent invasive modality capable of activating intracortical neuronal populations with high precision, enabling the fine manipulation of local neural circuits [10], and is developing into an effective tool for replacing or supplementing natural sensory inputs, probing and modulating dysfunctional neural circuits, and influencing behavior in experimental settings [11].

In early research, ICMS primarily served as an electrophysiological tool to map the functional topology of the cerebral cortex [12]. With the maturation of neural encoding and decoding technologies, ICMS has successfully induced stable and discriminable tactile [13] and visual [14] sensations in both animal models and human participants. Furthermore, it has been utilized as an auxiliary channel for information injection to guide the behavioral responses of animals [11]. This capability to inject information directly into the cortex makes ICMS an important foundation for sensory feedback in BCIs and indicates that its perceptual quality and behavioral utility are closely related to stimulation parameters, perceptual stability, and user-specific neural responses.

In recent years, studies have also begun to examine whether ICMS can bias neural connectivity over longer time scales. Specific temporal patterns or activity-dependent ICMS sequences can induce plasticity-like changes within cortical networks in selected paradigms, suggesting that ICMS may modulate synaptic connection strength and cortical representations and may be beneficial for selected neurological diseases [15]. This direction extends ICMS from transient cortical activation toward circuit-level neuromodulation and brings timing rules, activity-specific biomarkers, closed-loop control, and the durability and safety of induced plasticity into closer focus.

The above ICMS applications also depend on the stability of the implanted interface. To overcome physical limitations of conventional electrodes, including foreign body responses (FBRs), biohybrid neural interfaces (BNIs) that integrate living cellular components with electrodes provide new possibilities for improving tissue–device compatibility and supporting graft–host interactions, indicating that interface safety, controllability, and long-term functional stability require focused evaluation in future ICMS development [16].

**Fig. 1.**
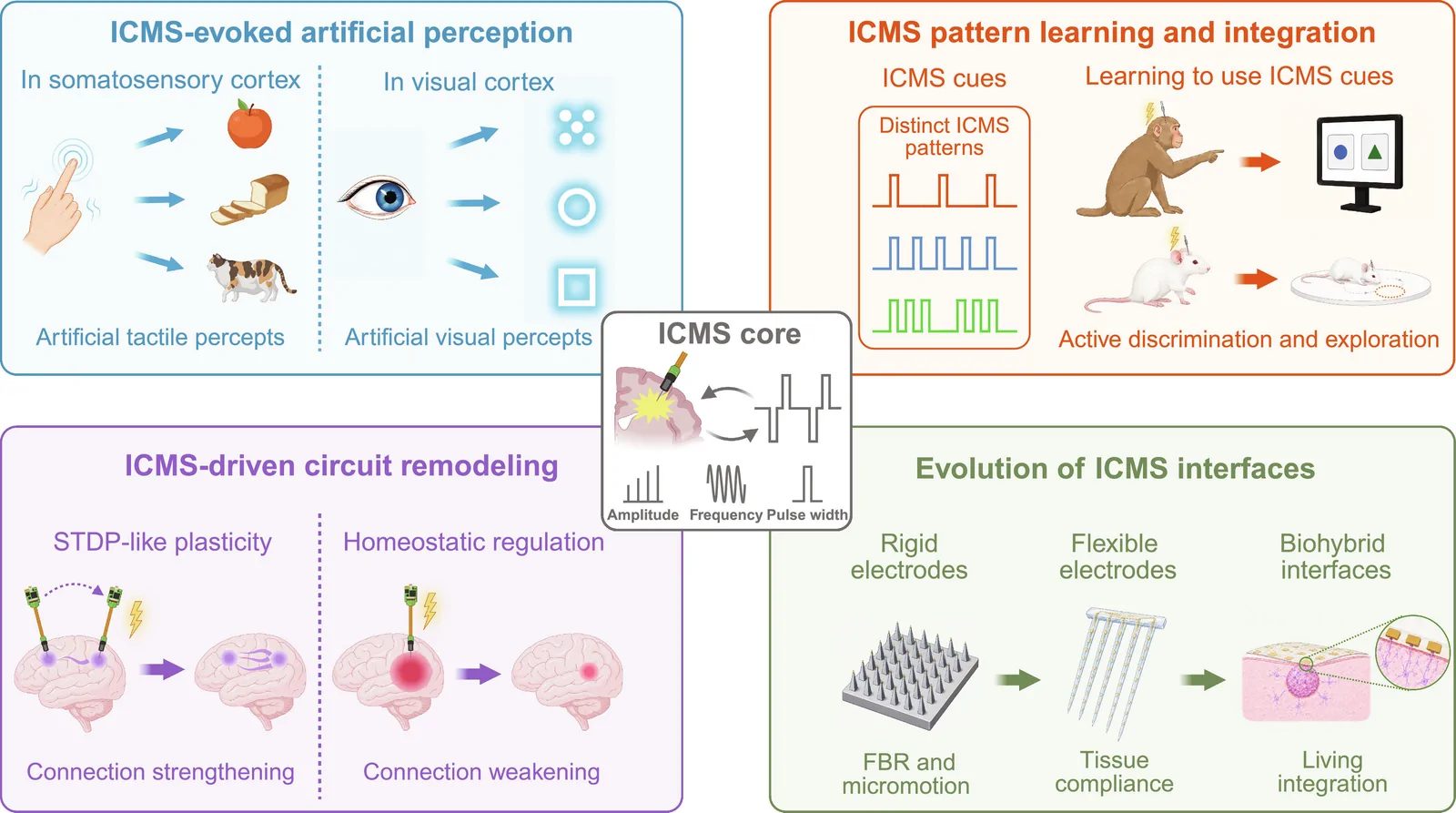
Conceptual framework for ICMS in BCIs. The schematic provides an overview of the major domains covered in this review: ICMS-evoked artificial perception, the learned use and integration of ICMS patterns, plasticity-related circuit remodeling, and the evolution of rigid, flexible, and biohybrid interfaces, centered on ICMS delivery and pulse parameters.

Here, we propose an integrative framework for ICMS applications in BCIs, organized around hardware interfaces, stimulation parameters, artificial perception/information input, plasticity-based modulation, biohybrid integration, and translational challenges. This framework is not limited to a single device or application. Rather, it explains how ICMS has progressed from transient cortical activation toward parameterized stimulation design, artificial information input, closed-loop plasticity modulation, and biointegrated interface exploration, while identifying limitations in long-term stability, mechanistic interpretation, safety, and scalability for its application in BCIs (Fig. 1).

## Technical Foundations: Microelectrode Interfaces and ICMS Parameters

**Fig. 2.**
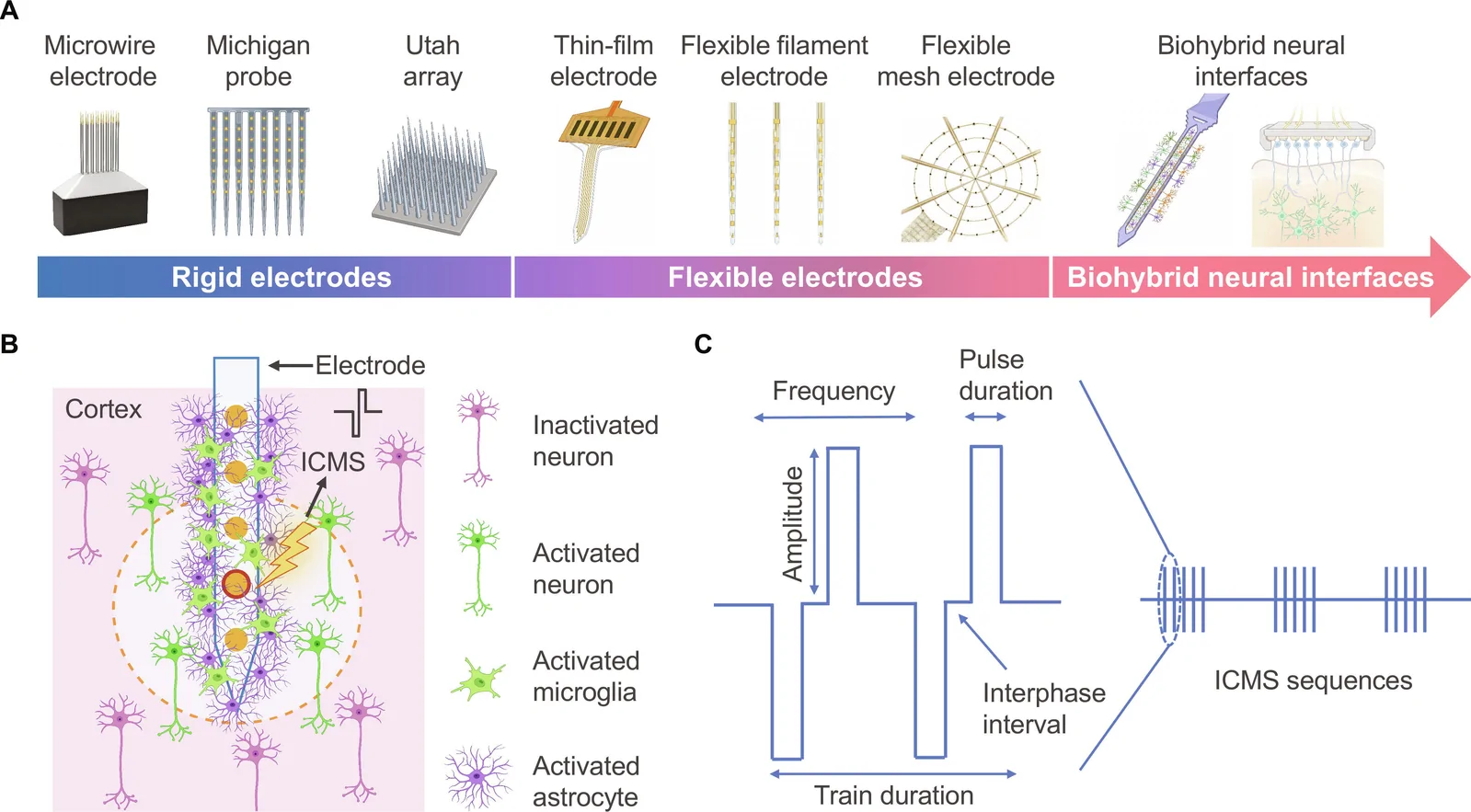
Evolution of microelectrode interfaces, FBR, and fundamental parameters of ICMS. (A) Schematic of the transition from rigid to flexible electrodes and biohybrid neural interfaces. Left to right: microwire electrode, Michigan probe, Utah array, thin-film electrode, flexible filament electrode, flexible mesh electrode, and biohybrid neural interfaces. (B) Rigid electrodes typically trigger FBR and glial scar encapsulation. During operation, ICMS activates adjacent neuronal populations via the exposed electrode contacts. (C) Typical parameters of an ICMS pulse train, including stimulation amplitude, frequency, pulse duration, interphase interval, and train duration.

ICMS operates primarily by directly delivering electrical pulses to brain tissue via implantable microelectrode arrays (MEAs) [17]. The stimulation sequence generally consists of μA-level, biphasic, cathodic-first pulses, with a charge-balanced design to minimize tissue damage while enabling the sparse activation of nearby neurons at relatively low amplitudes [10]. To meet the demands for chronic in vivo application, invasive MEAs are currently undergoing a paradigm shift: from rigid to flexible [18], from single-function to multi-function, from standard array structures to biomimetic architectures, and ultimately toward BNIs [19]. These advancements aim to provide chronic, stable, and minimally invasive electrode interfaces (Fig. 2A).

### Microelectrode interfaces

#### Rigid electrodes

Current ICMS research predominantly employs microwire or silicon-based electrodes. Microwire electrodes consist of micrometer-diameter filaments typically fabricated from materials such as platinum, iridium, platinum/iridium alloys, gold, tungsten, or stainless steel. These filaments are coated with an insulating layer, leaving only the tips exposed as recording or stimulation sites [20]. While these electrodes feature a straightforward design and low cost, their limited coating compatibility and durability render them unsuitable for the rigorous demands of chronic, high-frequency stimulation [21]. Silicon-based MEAs emerged from the development of microelectromechanical systems (MEMS) technology and provide an alternative to microwire-based MEAs [22–24]. Structurally, these are primarily classified as Michigan probes and Utah arrays. Michigan probes generally use thin shank-like structures with contacts arranged along the electrode length, allowing recording and stimulation across cortical layers, whereas Utah arrays use needle-like 3-dimensional (3D) structures with contacts near the tips to cover broader cortical regions [25,26]. These designs achieve multicontact integration, enabling simultaneous signal recording and precise stimulation across multiple cortical regions; when combined with micrometer/nanometer-scale precision surface etching and high-charge-capacity coatings such as sputtered iridium oxide film, they can substantially enhance durability, signal fidelity, and stimulation capabilities [22].

However, metal microwires (~100 to 300 GPa) and silicon (~180 GPa) possess high Young’s moduli [18], resulting in a profound mechanical mismatch with the exceptionally soft brain tissue (~1 to 30 kPa) [27]. This discrepancy makes them prone to causing micromotion-induced tissue damage and FBR, triggering neuroinflammation and glial scar encapsulation [28] (Fig. 2B). Consequently, this hinders the establishment of stable, chronic interactions between the electrode interface and local neurons [29]. As the dominant tools for in vivo experiments, these electrodes have undergone extensive chronic performance evaluations in animal models and a subset of human patients [30,31]. Although compensatory strategies such as ultraflexible cable interconnects [32] and bioactive surface modification [33] have been proposed, such strategies can partially improve interface performance but cannot eliminate the bulk mechanical mismatch between stiff implants and soft brain tissue; the interactions between microelectrodes and brain tissue remain exceedingly complex, and the challenges surrounding their chronic applicability still demand further resolution.

#### Flexible electrodes

To meet the demands for high-quality modulation and chronic implantation, microelectrode technology is advancing toward flexible platforms. Some flexible electrodes can be discussed within a broader biomimetic electronics framework, which aims to reduce micromotion-induced mechanical mismatch and FBR through material mechanics, device geometry, and tissue-compliant design [19,34]. Structurally, current flexible electrodes generally fall into 3 categories: flexible thin-film electrodes [35], flexible filament electrodes [36,37], and flexible mesh electrodes [38]. Utilizing insulating polymers (such as polydimethylsiloxane [PDMS], Parylene C, SU-8, polyimide, and polyethylene terephthalate) as flexible substrates provides a Young’s modulus that closely matches that of brain tissue. This effectively reduces mechanical damage and neuroinflammatory responses [37,39], enhances the long-term stability of the electrodes, and may reduce glial encapsulation and improve tissue integration in selected experimental settings. To overcome the high interfacial impedance caused by electrode miniaturization, conducting polymers such as poly(3,4-ethylenedioxythiophene):polystyrene sulfonate and polypyrrole (PPy) can be introduced. These polymers enhance the electrochemical performance of the electrodes and reduce the ICMS charge injection threshold, thereby providing a favorable microenvironment for cell adhesion and synaptic growth [40].

To further improve interfacial biocompatibility, flexible electrodes can also incorporate bioactive surface modification strategies. By incorporating specific extracellular matrix proteins, such as laminin, anti-inflammatory peptides, or defined cell adhesion molecules onto flexible material surfaces or within hydrogel coatings, these strategies create a highly biomimetic local microenvironment that supports host neuronal adhesion and synaptic growth [41]. This approach can complement the mechanical advantages of flexible electrodes, but current progress should still be interpreted cautiously. Flexibility itself entails engineering constraints in chronic insulation and encapsulation stability, precise implantation, stimulation safety windows, and consistency in high-throughput manufacturing. Therefore, reduced impedance, improved histology, or enhanced cell adhesion alone is insufficient to demonstrate long-term, stable, and scalable ICMS application across conditions and species [18,42]. For flexible electrodes to become a complete solution for long-term ICMS, further clinical and functional validation in implanted systems, such as the Neuralink platform, is still needed [36]. These limitations also suggest that subsequent interface design should move beyond simple mechanical matching toward regulation of tissue–device interactions.

#### Biohybrid neural interfaces

While flexible substrates and bioactive surface modification strategies have improved electrode biocompatibility, they still cannot fully bridge the fundamental barrier between artificial devices and biological tissues [41,43]. To address this limitation, researchers are attempting to construct new BNIs based on biohybrid electronics. This concept extends bioactive surface modification by introducing living biological components into the tissue–device interface to mediate interactions between electronic devices and the nervous system and to enhance biocompatibility and tissue integration [19,44,45]. Such interfaces have been shown to effectively reduce electrode-implantation-induced FBR and promote closer integration between the electrode interface and the cerebral cortex [46].

Further, biohybrid regenerative electronics emphasizes regenerative-medicine and tissue-repair goals. BNIs constructed from biological components such as neural stem cells (NSCs) or brain organoids may allow ICMS to participate in graft development and integration, and may benefit the repair or reconstruction of injured tissue by monitoring and modulating graft function [47]. This strategy may provide new application routes for ICMS in traumatic brain injury (TBI) and other neurological diseases, but its ethical and safety constraints require further consideration [48].

The above ICMS-oriented microelectrode-interface progress has been conceptualized as an evolution from “biomimetic and bioactive electronics” to “biohybrid electronics” and ultimately to “living electronics and interfaces”, and may provide a more advanced hardware basis for chronic ICMS applications while expanding its application scope [19].

### ICMS pulse-train parameters and neuronal recruitment

ICMS is not simply the delivery of electrical pulses to the cortex. Electrode structure, contact location, and pulse-train parameters all influence which neural elements are recruited and what functional effects follow. Microampere-level extracellular pulses can recruit axons, somata, dendrites, and trans-synaptic pathways. Axonal activation is central to spatial spread [10,49–51]. Early studies proposed that ICMS forms an approximately spherical effective activation zone around the electrode tip and can be described by the *I* = *kr*^2^ current–distance relationship, where *I* is current, *r* is distance from the electrode, and *k* is the current–distance constant [52]. Subsequent imaging and modeling studies further indicate that ICMS is better understood as a sparse, distributed, and parameter-dependent recruitment process: increasing stimulation amplitude, pulse width, or delivered charge can enhance axonal recruitment and trans-synaptic effects, but the spatial extent of activation is also shaped by axonal orientation, cell type, and local circuit organization [10,53].

Common ICMS protocols use charge-balanced biphasic pulses, often cathodic-first, to limit net charge accumulation and tissue damage while enabling neural activation [54,55]. The key variables are stimulation amplitude, frequency, pulse duration, interphase interval, and pulse-train duration [56] (Fig. 2C). These variables jointly influence neural recruitment, perceptual effects, adaptation, and safety boundaries [10,57,58]. These effects are not simply linear: stimulation amplitude and pulse width mainly influence recruitment strength and spatial spread, whereas frequency and interpulse interval further shape short-latency excitation, prolonged inhibition, and pulse-to-pulse interactions. Therefore, the same parameters may produce different recruitment outcomes across electrode sites, cortical layers, or tissue states [59].

Thus, a single optimal ICMS parameter combination is unlikely to generalize across applications. Parameter differences reflect electrode-contact location, tissue state, stimulation target, perceptual modality, and task demands, as well as application-specific requirements for recruitment range, temporal structure, and safety boundaries [11,60]. For example, tactile studies emphasize perceived intensity, naturalness, and localization [13,61–65]; visual studies focus on phosphene location, brightness, discriminability, and shape perception [14,66,67]; learnable-information studies test whether temporal, spatial, or sensor-coupled ICMS patterns can be learned and integrated with natural sensory information [68–74]; and plasticity-induction studies rely more strongly on millisecond-scale timing between spontaneous neural activity and stimulation, with spike-triggered activity-dependent ICMS often using low-frequency, single-pulse triggering [15,75–78]. Table 1 summarizes representative studies by application category, species range, electrode type, major pulse-train parameter ranges, and experimental duration range.

**Table 1.** Representative ICMS pulse-train parameters across major application categories

Category	Species range	Electrode type	Amplitude	Frequency	Pulse width	Interphase interval	Train duration	Experimental duration range
Tactile perception [13,61–64,88,89]	Human	Utah arrays	2–100 μA	0–300 Hz	~200 μs/phase	53–100 μs	0.05–3 s (or real-time driven)	1 d–84 mo (NR in some)
Visual perception [14,66,67,91,204]	Human/Nonhuman primates	Microwire electrodes/Utah arrays	1–300 μA	50–300 Hz	100–800 μs/phase	0–100 μs	0.125–3 s	4–24 mo (NR in some)
Learnable information input [68–74,94,95]	Nonhuman primates/Rodents	Microwire electrodes/Utah arrays	1–300 μA	0–400 Hz or event-triggered	100–200 μs/phase	25–250 μs	50 ms–5 s (or real-time driven)	3 d–4 mo (NR in some)
Plasticity modulation [15,75–78,115,117–121]	Nonhuman primates/Rodents	Microwire electrodes/Michigan probes/Utah arrays	20–330 μA	Spike-triggered single pulse (~7–19 Hz) or 300–1,000 Hz (paired stimulation)	192–200 μs/phase	NR	Single-pulse stimulation or ~2.8–6.7 ms (paired stimulation)	0.5 h–21 d

NR, not reported; h, hours; d, days; mo, months

In parameter selection, stronger, longer, or higher-frequency stimulation can enhance neural recruitment and behavioral effects, but may also increase threshold drift, tissue responses, chronic interface deterioration, neuronal loss, or myelinated fiber atrophy [79–81]. Therefore, Table 1 should be understood as summarizing representative choices rather than universal “safe ranges”. Species differences, application category, electrode interface, and experimental duration can all alter the trade-off between safety and stimulation effects, and parameter translation across studies should be calibrated with cross-species functional equivalence and computational models, with attention to the recruited cortical layers, neuronal populations, and functional outcomes [60,82].

Together, these findings suggest that future ICMS parameter optimization should move beyond empirical tuning toward task-specific, individualized, and potentially closed-loop strategies that account for circuit recruitment, perceptual stability, adaptation, and long-term tissue responses [60]. Based on diverse electrode platforms and parameter combinations, ICMS has made important progress, particularly in artificial tactile and visual perception.

## Artificial Perception and Neural Information Injection

Initially, ICMS was primarily used to determine the functional representations of different cortical areas [12]. For example, activating the motor cortex elicits repetitive muscle movements [83], enabling the generation of precise functional cortical maps [84]. Recently, this approach has transitioned from fixed, simple stimulation sequences to high-frequency, long-duration, and complex patterns, revealing more intricate motor information encoding [85]. Furthermore, it can define effective intracortical connectivity networks via cortico-cortical evoked potentials (CCEPs) and stimulation-evoked effective connectivity (SEEC) [86,87], holding promise as a novel method for intraoperative functional cortical mapping.

As research has progressed, ICMS has been increasingly applied to the development of sensory neuroprostheses in BCIs. In this context, the clinical goal of sensory restoration is essentially achieved by evoking artificial perception. Delivering ICMS to precise locations within the primary somatosensory cortex (S1) or primary visual cortex (V1) can induce diverse, transient perceptual experiences in primates and humans [13,14]. Establishing tactile or visual feedback via ICMS in BCIs may not only improve the psychological well-being of patients but also enhance motor task performance [11,88]. With advancements in neural encoding research, ICMS-evoked responses have become more defined and controllable, making ICMS-based artificial perception promising [14,61–64,66]. Next, we discuss how ICMS can evoke artificial perception and provide learnable information input, while noting limitations such as parameter dependence and perceptual stability.

### Evoking artificial tactile and visual perception

**Fig. 3.**
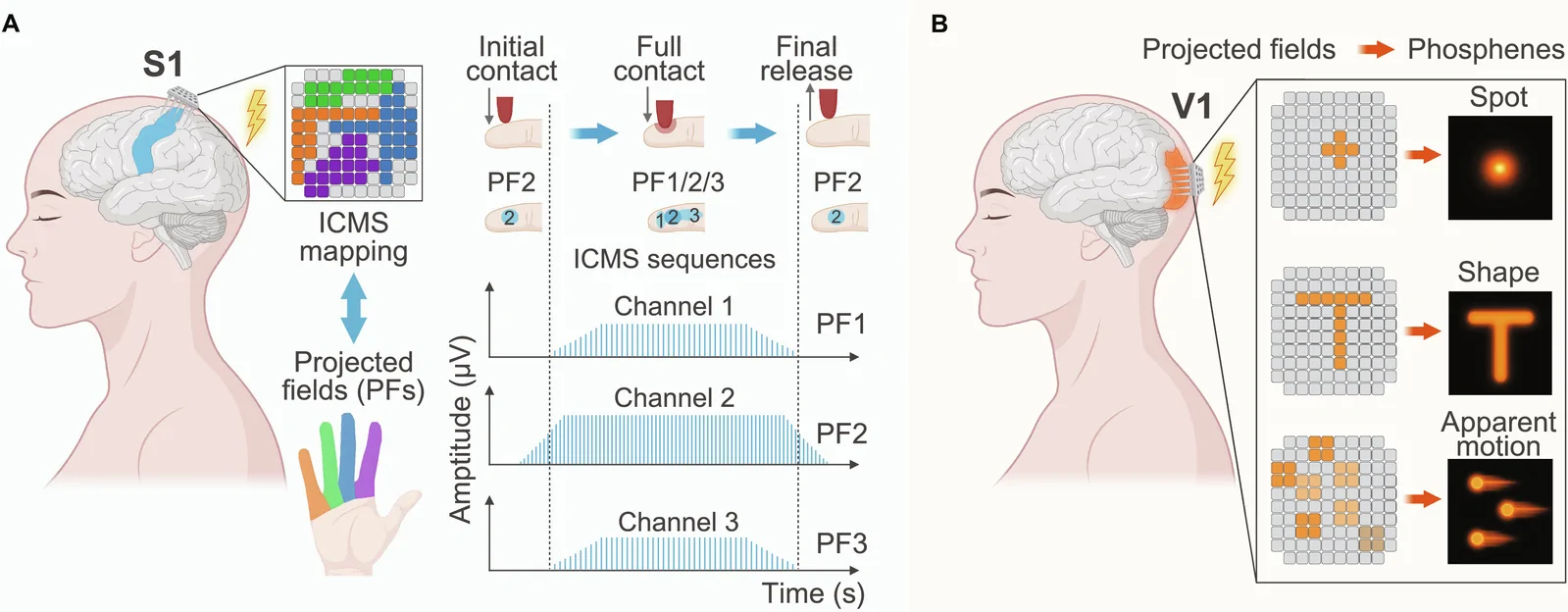
Artificial perception paradigms in the primary somatosensory and visual cortices via ICMS. (A) In S1, ICMS evokes tactile perception based on the spatial correspondence between RFs and PFs. Spatiotemporally patterned ICMS coordinates stimulation amplitude and timing across channels to convey tactile edge curvature and apparent motion. (B) In V1, ICMS leverages the retinotopic organization of RFs to evoke phosphenes, constructing complex visual percepts, such as spots, shapes, and directional apparent motion, through patterned multielectrode stimulation.

ICMS-evoked artificial perception is not equivalent to a complete reconstruction of natural sensory activity, but is better understood as a cortical input that can be localized, modulated, and learned. Receptive fields (RFs) refer to body or skin regions where natural peripheral stimuli can evoke responses from a specific neuronal population or cortical site [12]. Based on these S1 RFs, ICMS can evoke tactile sensations (such as numbness, tingling, or pressure) in the corresponding areas [8,12], with perceptual intensity generally correlating positively with stimulation amplitude and frequency, which provides the basis for linear encoding that maps a single stimulation parameter onto perceptual intensity [13,65]. In this process, projected fields (PFs) refer to the body regions where participants report the sensation after stimulation of a specific cortical site, and usually correspond to or fall within the spatial extent of that site’s RFs. Based on this RF–PF relationship, ICMS can evoke spatially adjustable localized tactile percepts and support parameterized tactile encoding [89] (Fig. 3A). However, interactions among amplitude, frequency, electrode combination, and other parameters may be nonlinear, increasing variability in stimulation effects and reducing the feasibility of multiparameter linear encoding and parameter transfer [65,90].

Overall, early S1 studies established the feasibility of localized artificial touch, whereas later human studies have shifted toward multielectrode combinations and more complex tactile encoding. Multielectrode ICMS is a spatial delivery strategy that distributes stimulation across multiple electrode contacts, which can reduce charge input at individual contacts and may improve perceptual spatial resolution or PF stability [8]. Subsequent human BCI work showed that multielectrode ICMS-evoked tactile percepts can remain relatively stable, that their intensity can be modulated by stimulation amplitude, and that they can be linked to prosthetic-hand sensor signals to provide tactile feedback in bidirectional BCIs. This feedback, however, still mainly relies on approaches such as linear amplitude modulation and provides basic tactile feedback without fully resolving perceptual naturalness [13,61,88].

Beyond spatial-distribution strategies, biomimetic ICMS places greater emphasis on stimulation timing and dynamic parameter design, aiming to approximate population-level neural responses evoked by natural sensory inputs by adjusting pulse amplitude, frequency, or spatiotemporal patterns rather than encoding a single sensory variable with a single parameter [8]. In natural touch, when making or breaking contact with an object, mechanoreceptors in the skin generate a high-frequency transient firing known as a “contact transient”, whereas the firing frequency decreases during sustained pressure. The biomimetic patterning of ICMS leverages this phenomenon by delivering a brief, high-amplitude or high-frequency burst at the onset of stimulation before reducing it to a lower baseline level. Compared to conventional linearly encoded ICMS, where the amplitude increases linearly with pressure, biomimetically encoded ICMS significantly improves the subjects’ tactile resolution, resulting in a lower just-noticeable difference (JND) and enhancing the naturalness and dynamic intensity range of the touch [61,63]. Spatiotemporally patterned ICMS further builds on multielectrode ICMS by coordinating electrode combinations in space with stimulation sequence, frequency, and amplitude changes in time. By using voltage field shaping (VFS) to dynamically adjust the amplitude ratios across electrodes, it can evoke clear perceptions of edges, curvature, and continuous motion [62]. More recent personalized and interactive ICMS paradigms allow participants to actively adjust amplitude, frequency, the biomimetic degree of stimulation encoding, and temporal overlap during sequential electrode activation, and can establish highly specific virtual-object perceptual models to convey complex tactile features such as object texture and compliance [64]. Together, these results support the extension of ICMS from linear intensity encoding to multielectrode, biomimetic, and more complex spatiotemporal pattern design. These strategies improve spatial delivery, temporal dynamics, or subjective controllability in different ways, but they still mainly enhance naturalness and controllability at the perceptual-outcome level and do not yet approximate the population-level activity patterns of natural tactile input [8].

When applied to V1, ICMS can induce phosphenes (such as spots or lines of light), with brightness, shape, spatial location, and discriminability closely related to current amplitude, pulse timing, electrode combination, and cortical location [14,67,91,92]. Utilizing the specific spatial arrangements of V1 RFs and the retinotopic map, spatiotemporally patterned ICMS can construct recognizable static letters (e.g., T and L) and directional apparent motion within the visual field of monkeys. This recognition ability can be directly acquired during visual tasks and transferred to ICMS-evoked phosphene patterns, demonstrating that high-channel-count ICMS can generate spatially structured phosphene patterns across visual cortex [14]. In similar experiments involving completely blind human participants, synchronized patterned multielectrode stimulation successfully evoked simple 2-dimensional (2D) shapes and letters distinguishable by the subjects, and a closed-loop system incorporating a bio-inspired retinal encoder allowed subjects to locate external objects via head scanning [66]. These findings indicate that visual ICMS has progressed from single-phosphene induction toward structured visual-cue construction (Fig. 3B). Current evidence, however, still mainly supports simple shapes, letters, and object localization. Further development remains limited by phosphene predictability, multielectrode combination rules, retinotopic mapping, and chronic stability, and long-term stable, generalizable image perception has not yet been achieved [8,14,66,67].

In summary, multiple complex stimulation strategies have advanced ICMS-based artificial perception from single-site evocation toward greater controllability, but their advantages and limitations differ. Multielectrode ICMS expands the spatial distribution of stimulation and can improve spatial controllability, while remaining influenced by electrode layout, interchannel interactions, and cortical layers. Biomimetic ICMS currently has application evidence mainly from tactile studies and can improve tactile discrimination and subjective naturalness, but its stability and transferability remain to be validated. Spatiotemporally patterned ICMS further integrates electrode combinations with stimulation timing and can convey more complex structured artificial perceptual cues in touch and vision, but it remains limited by stimulation-combination rules and chronic stability [13,14,61–64,66,67,88,93]. Therefore, the translational limitations of ICMS arise not only from stimulation-pattern design but also from its mode of neural recruitment. Electrical stimulation can bypass normal synaptic integration and generate neuronal activation patterns that differ from natural sensory input. Continuous stimulation can also cause rapid perceptual fading during stimulation and longer-lasting stimulation-induced depression of neuronal excitability. In human tactile ICMS, perceptual effects may fade in less than 1 min during continuous stimulation, which is an important obstacle to sustained feedback under clinical conditions [8]. Representative parameters for the tactile and visual ICMS studies discussed here are summarized in Table 2.

**Table 2.** Representative stimulation parameters and electrode configurations in tactile and visual ICMS studies

Study	Species	Target area	Electrode	Amplitude	Frequency	Pulse width	Interphase interval	Train duration	Experimental duration	Main findings
[13]	Human (*N* = 1)	S1	Utah	10–100 μA	25–300 Hz	200 μs cathodic, 400 μs anodic	100 μs	0.5–1 s	6 mo	S1 ICMS evoked stable localized touch
[89]	Human (*N* = 1)	S1	Utah	20–100 μA	50–300 Hz	200 μs per phase	53 μs	1 s	3–56 d	S1 ICMS evoked cutaneous/proprioceptive percepts
[88]	Human (*N* = 1)	S1; M1 (recording)	Utah	14–64 μA	Average 100 Hz	200 μs cathodic, 400 μs anodic	100 μs	NR	23 mo	S1 ICMS tactile feedback improved BCI control
[61]	Human (*N* = 3)	S1; M1 (recording)	Utah	20–90 μA	50–200 Hz	200 μs cathodic, 400 μs anodic	100 μs	0.1/1 s	24–84 mo	Multielectrode/biomimetic ICMS supported stable precise PFs
[63]	Human (*N* = 3)	S1	Utah	2–80 μA	0–250 Hz	200 μs cathodic, 400 μs anodic	100 μs	1 s	1–3 d	Biomimetic ICMS enhanced natural touch at lower charge
[64]	Human (*N* = 3)	S1	Utah	10–100 μA	20–150 Hz	200 μs cathodic, 400 μs anodic	100 μs	NR	NR	Customized ICMS evoked object-specific touch
[62]	Human (*N* = 2)	S1; M1 (recording)	Utah	40–90 μA	100 Hz	200 μs cathodic, 400 μs anodic	100 μs	0.05–3 s	NR	Patterned ICMS evoked edges, curvature, and motion
[67]	Human (*N* = 1)	V1	Microwire	1.9–80 μA	50–200 Hz	100–800 μs per phase	0–100 μs	0.125–3 s	4 mo	V1 ICMS evoked stable phosphenes
[204]	Macaque monkey (*N* = 1)	V1	Microwire	12–25 μA	200 Hz	200 μs per phase	NR	0.2–1 s	16 mo	V1 ICMS supported phosphene localization
[91]	Macaque monkey (*N* = 1)	V1	Utah	18–300 μA	200 Hz	200 μs per phase	100 μs	200 ms	19–24 mo	Chronic V1 microstimulation supported spatial discrimination
[14]	Macaque monkey (*N* = 2)	V1; V4 (recording)	Utah	1–210 μA	300 Hz	170 μs per phase	60 μs	167/450 ms	NR	Multielectrode V1 ICMS supported simple pattern recognition
[66]	Human (*N* = 1)	V1/V2 boundary	Utah	1–128 μA	300 Hz	100–800 μs per phase	60 μs	166 ms	6 mo	Occipital ICMS evoked phosphenes and simple patterns

NR, not reported; M1, primary motor cortex; V2/V4, visual cortical areas V2/V4; h, hours; d, days; mo, months

### ICMS pattern learning and information integration

**Fig. 4.**
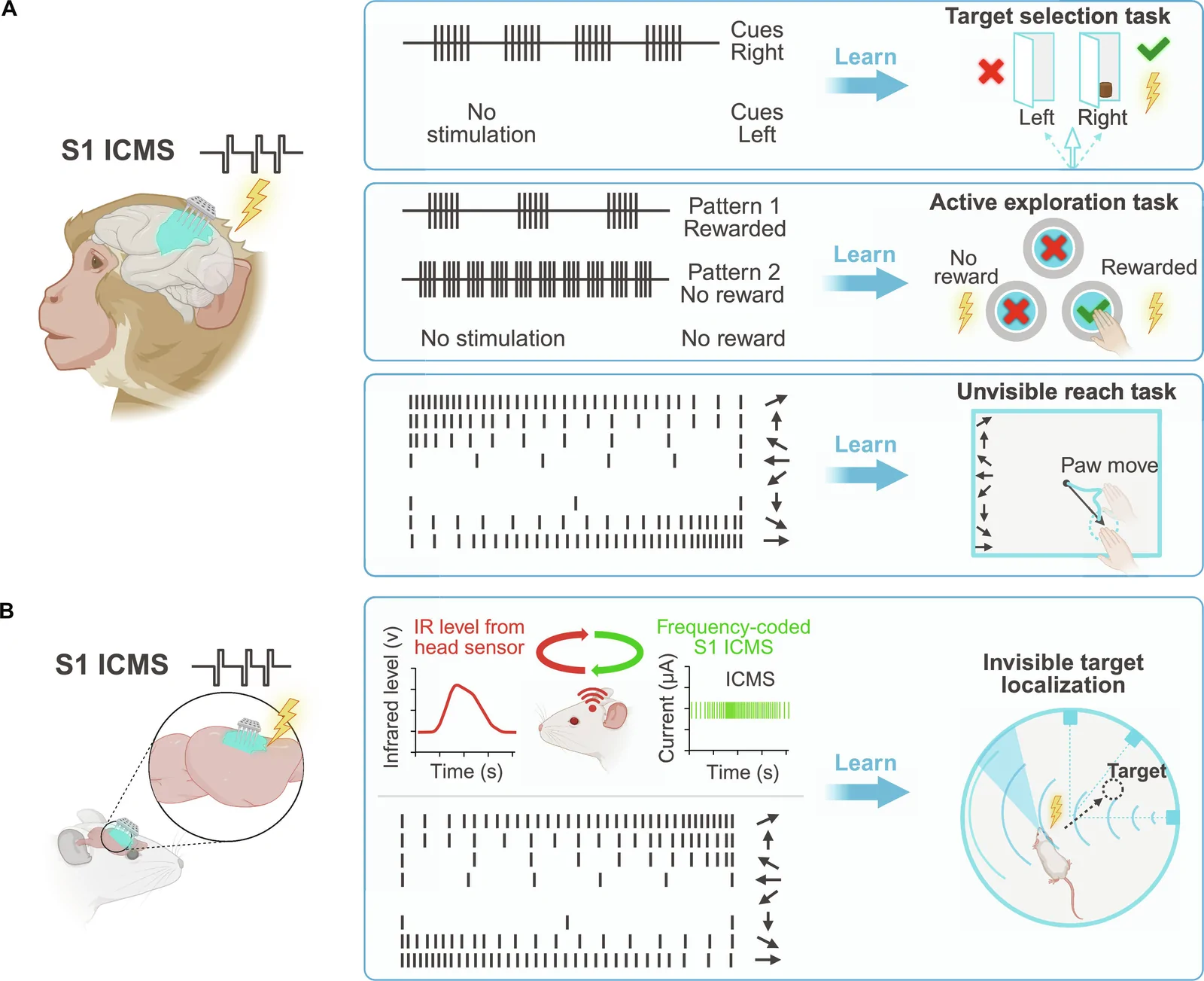
ICMS pattern learning and multisensory information integration. (A) Following training, primates learn to interpret ICMS sequences with diverse spatiotemporal patterns to execute target selection, active exploration, and reaching toward invisible targets. (B) Rodents learn to use sensor- or behavior-coupled ICMS to localize and explore invisible targets. Infrared signals can be mapped onto S1 stimulation frequency, whereas patterned multichannel ICMS can distribute directional and distance-related information across electrodes.

Distinct from the percept-evocation strategies discussed above, another direction of research treats ICMS as meaningful, learnable signals. A classical paradigm involves training primates to learn and discriminate different spatial or temporal patterns of ICMS sequences delivered to the sensorimotor cortex, prompting them to perform corresponding behaviors to obtain rewards (Fig. 4A). For instance, owl monkeys successfully learned to discriminate 4 distinct spatial or temporal ICMS patterns applied to the hand representation area of S1, using these cues to locate food hidden behind either a left or right door [68]. Rhesus macaques were able to complete 3 distinct visual tasks: center-out reaching, continuous target tracking, and target selection, which were cued either by mechanical vibration on the hand or by ICMS within S1 [69]. Furthermore, during active exploration, delivering specific, reward-associated ICMS patterns to S1 enabled monkeys to learn to distinguish between 3 visually identical targets [70], or to discriminate texture coarseness conveyed by different invisible virtual gratings [94]. These studies show that, after training, ICMS can become an actionable cue for discrimination, reaching, and active exploration, rather than a direct reproduction of natural sensory encoding or task-related neural activity. Notably, in a control experiment, posterior parietal cortex stimulation failed to form an effective behavioral cue, suggesting that this form of information input is target dependent and may fundamentally rely on distinguishable somatic sensations evoked by stimulation [70].

In addition, animals have also been shown to learn to integrate ICMS with visual cues to explore invisible targets (Fig. 4B). In rhesus macaques, delivering continuous spatial information via 8-channel ICMS to S1 to gradually replace visual signal (VIS) cues allowed the monkeys to learn a center-out reaching task toward an invisible target [74]. In rats, using S1 ICMS frequency as a single coding variable that changed continuously with behaviorally relevant variables such as the angle or distance to a target could also replace VIS cues. Through active exploration of invisible locations, rats learned to obtain water rewards or complete water-maze search tasks, while still distinguishing artificial input from native whisker deflections [71,72]. Subsequent work extended this coding approach to 2 dimensions: multielectrode ICMS encoded target angle and distance in real time through stimulation frequency and electrode channel, allowing mice to replace VIS cues, integrate the artificial input with natural vision, and complete a 3 × 3 grid-navigation task with high accuracy [73]. Additionally, gradually substituting visual cues with ICMS applied to the dorsal premotor cortex trained rhesus macaques to discriminate ICMS patterns across different electrode contact combinations, enabling highly accurate judgments in a 4-class reach-and-grasp task [95]. Interestingly, in some studies, combined VIS+ICMS cueing produced higher accuracy than VIS-only or ICMS-only cueing [73,74], and native tactile representations were not disrupted [71,72]. This suggests that ICMS-carried information can remain partly independent from existing tactile representations while being incorporated into other sensory modalities in a naturalistic manner.

These studies suggest that ICMS does not necessarily have to perfectly replicate natural neural encoding to be effective. Instead, the brain can treat it as a novel sensory modality or an abstract instruction and, through learning, naturally comprehend these task cues or integrate them with other natural sensory modalities, thereby expanding or augmenting natural perception [11]. This is informative for expanding the roles and contexts of ICMS in BCI feedback, but these effects mainly arise in structured tasks with explicit training, reward mappings, and constrained behavioral goals. Learnable ICMS is therefore better interpreted as a task-dependent information channel than as a universally transferable sensory code. Recently, ICMS informed by the S1-to-primary motor cortex (M1) pathway significantly modulated grip force without prior associative training, suggesting that ICMS designed according to cortical information pathways may be more efficient [96]. Future work should compare cortical targets and training requirements, standardize training metrics, and test whether learned mappings remain stable across training stages and task modalities. Representative parameters for the learnable ICMS information-input paradigms discussed here are summarized in Table 3.

**Table 3.** Representative stimulation parameters and electrode configurations in studies of ICMS pattern learning and information integration

Study	Species	Target area	Electrode	Amplitude	Frequency	Pulse width	Interphase interval	Train duration	Experimental duration	Main findings
[68]	Owl monkey (*N* = 2)	S1	Microwire	50–200 μA	100 Hz	100 μs	100 μs	4 s	3–4 mo	Multichannel S1 ICMS supported reaching cues
[69]	Rhesus macaque (*N* = 1)	S1/PP; M1/PMd/SMA (recording)	Microwire	25–60 μA	30–60 Hz	150–200 μs per phase	100 μs	0.5–2 s	NR	S1 ICMS supported cue learning; PP ineffective
[70]	Rhesus macaque (*N* = 2)	S1; M1 (recording)	Microwire	80–200 μA	200 Hz or 400 Hz	105–200 μs per phase	25 μs	50 ms	NR	S1 ICMS supported active texture discrimination
[71]	Rat (*N* = 6)	S1	Microwire	1–300 μA	0–400 Hz, updated every 50 ms	100 μs	50 μs	0.6–1.5 s	Approximately 51 d	S1 ICMS served as an infrared sensory channel
[74]	Rhesus macaque (*N* = 2)	S1	Utah	30–80 μA	100–300 Hz	200 μs per phase	250 μs	0.2–1.5 s	NR	S1 ICMS supported artificial sensory-visual integration
[95]	Rhesus macaque (*N* = 2)	PM/S1; M1 (control)	Microwire	PM/S1:2–28/20–60 μA	20–100 Hz	200 μs per phase	NR	50–1,000 ms	NR	PM/S1 ICMS supported action instruction learning
[72]	Rat (*N* = 6)	S1; A1 (control)	Concentric bipolar electrode	15–75 μA	100 Hz	200 μs per phase	NR	500 ms	3–7 d	S1 ICMS guided active sensing
[94]	Rhesus macaque (*N* = 2)	S1; M1 (recording)	Microwire	130 μA	Event-triggered, no fixed frequency	100 μs per phase	NR	NR	NR	S1 ICMS supported virtual texture discrimination
[73]	Mouse (*N* = 10)	S1	Microwire	10–20 μA	0–200 Hz, real-time updating	200 μs per phase	250 μs	5 s	≥7 d	S1 ICMS supported visual integration during navigation

NR, not reported; A1, primary auditory cortex; PM, premotor cortex; PMd, dorsal premotor cortex; PP, posterior parietal cortex; SMA, supplementary motor area; h, hours; d, days; mo, months

### Cortical representation reorganization by chronic ICMS

During the process of mapping cortical functions or evoking tactile perception using ICMS, researchers observed a critical phenomenon: in rodents and primates, chronic, low-frequency, repetitive ICMS can induce sensorimotor representation areas to undergo sustained and reversible boundary expansion. This expansion is predominantly characterized by complete substitution, with only a small fraction overlapping with other functional areas, and it gradually recovers after the cessation of stimulation [97,98]. In seizure-kindling models, motor-map enlargement is also associated with enhanced synaptic and field-potential responses, suggesting that abnormal repetitive activity can reshape cortical maps through potentiation-like mechanisms [99]. This chronic stimulation also sensitized the brain’s perception of ICMS, causing the stimulation threshold required to evoke the same response to decrease over time [49]. This heightened sensitivity is likely related to the plastic adaptation of the cortex to ICMS [93]. The continuous delivery of paired ICMS sequences with a fixed temporal delay between 2 electrode contacts in the forelimb area of the rat S1 significantly reduced the rat’s perceptual threshold and gradually enhanced the inferred functional connectivity between the 2 stimulation sites, further supporting this hypothesis [75].

These studies reveal an important transition in the interpretation of ICMS, indicating that the brain can integrate ICMS into ongoing cortical activity and respond in a targeted manner, thereby going beyond mere perceptual evocation [11]. Repeated ICMS does not merely “passively activate” neural tissue to evoke transient percepts; rather, it may be incorporated into ongoing cortical dynamics, actively inducing alterations in cortical functional representations and dynamically adjusting neural connection strengths. The underlying mechanism may be consistent with the synaptic plasticity engaged during the brain’s motor learning processes [100]. These phenomena support the view that ICMS may move beyond transient artificial perception, but whether it can reliably produce neural connectivity remodeling still requires further discussion.

## Neural Connectivity Remodeling Based on Synaptic Plasticity

**Fig. 5.**
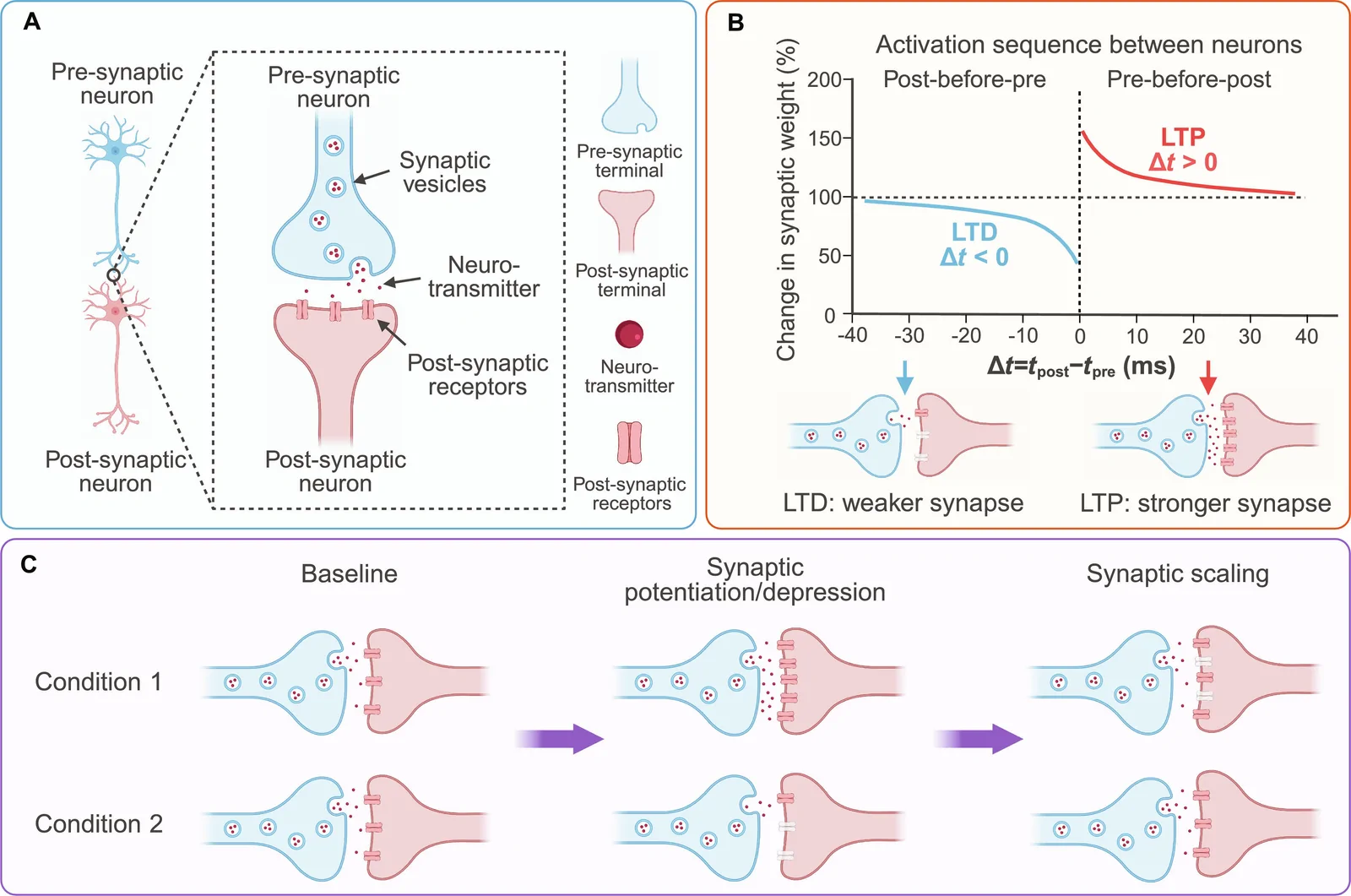
Synaptic mechanisms relevant to ICMS-induced plasticity. (A) Basic organization of chemical synaptic transmission. (B) STDP is a timing-dependent Hebbian mechanism: presynaptic activity preceding postsynaptic activity induces LTP, whereas the reverse sequence induces LTD. (C) Homeostatic synaptic scaling readjusts synaptic strength toward baseline after sustained increases or decreases in neuronal activity.

Beyond artificial perception, another key question is whether temporally structured electrical pulses can continue to influence cortical connectivity and network responses after stimulation ends. Synaptic plasticity, the ability of synaptic connections to change strength in response to neural activity, serves as the foundation for brain development [101] and postinjury functional reorganization [102], providing a key mechanistic framework for understanding how ICMS may induce longer-lasting network changes (Fig. 5A). Based on classic Hebbian plasticity [103], neurons that fire together tend to wire together, whereas poorly coordinated connections are weakened or become unstable; at the network level, these 2 effects are expressed as long-term potentiation (LTP) and long-term depression (LTD) [104]. LTP is generally induced by high-frequency, high-intensity stimulation, whereas LTD is generally induced by low-frequency, weak stimulation; both may be accompanied by physiological changes such as dendritic spine remodeling and altered local circuit excitability [105,106]. Spike-timing-dependent plasticity (STDP) further introduces a millisecond-scale temporal window and reveals a rule for synaptic weight change: a presynaptic pulse preceding a postsynaptic pulse tends to induce LTP, whereas the reverse order tends to induce LTD [107] (Fig. 5B). Burst-timing-dependent plasticity (BTDP) further extends this timing view by emphasizing that brief postsynaptic bursts paired with presynaptic input may produce stronger or more durable synaptic modification, although direct in vivo evidence for BTDP-like mechanisms in ICMS remains limited [108,109]. For ICMS, these plasticity rules are useful not because electrical stimulation reproduces natural physiological processes in full, but because they define timing constraints under which artificial pulses may bias synaptic efficacy.

Traditionally, reorganizing cortical representations and synaptic connections relies on chronic natural inputs, such as physical tactile [110] or visual [111] stimulation, or continuous motor skill learning [112]. ICMS, however, because of its intracortical nature, may “bypass” peripheral receptors. By delivering electrical pulses with specific timing, ICMS can simulate the neural activity required for synaptic enhancement and induce STDP-like connectivity changes within cortical networks [15,76,77]. This feature helps distinguish ICMS from neuromodulation approaches that primarily act on peripheral nerves, cortical surfaces, or deep nuclei: ICMS does not primarily alter network excitability at a larger scale, but, under appropriate experimental conditions, introduces precisely timed activity into defined intracortical microcircuits [9,11,113].

### Targeted connectivity modulation and damaged circuit repair

**Fig. 6.**
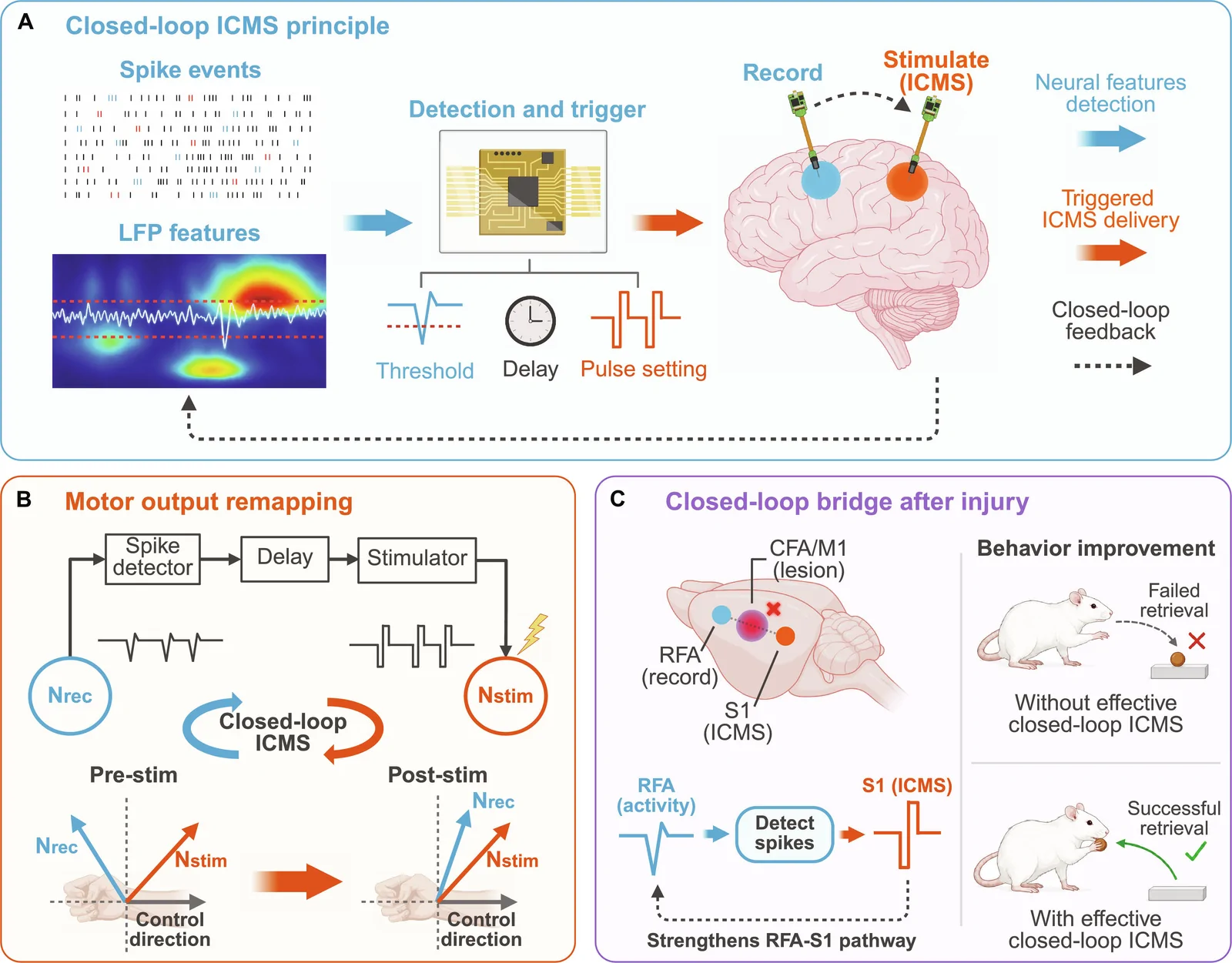
Closed-loop ICMS paradigms and circuit remodeling. (A) In closed-loop ICMS, spike events or LFP features detected from ongoing recordings trigger stimulation according to predefined thresholds, delays, and pulse settings. Neural activity after stimulation is then recorded as feedback for the next detection cycle. (B) An ADS paradigm detects spikes at one cortical site and delivers ICMS at another after a predefined delay. Repeated pairing can guide a directional shift in cortical motor output representations. (C) In rodents with M1 lesions, a closed-loop bridge uses activity recorded from RFA to trigger S1 ICMS, strengthening the RFA-S1 pathway and improving motor recovery.

Current ICMS research is shifting from open-loop stimulation (i.e., paired stimulation sequences with a fixed temporal delay between microelectrode contacts) [75] toward plasticity-based closed-loop modulation. This approach, commonly implemented through activity-dependent stimulation (ADS), involves the real-time detection of spontaneous neural activity in a recording area, such as spikes or specific band power of local field potentials (LFPs), followed by the delivery of ICMS to a target area after a millisecond-scale delay, thereby artificially strengthening the functional connection between the 2 regions [114] (Fig. 6A). Compared with conventional paired stimulation, ADS is more informative mechanistically because the pulse is contingent on ongoing circuit activity rather than imposed independently of the circuit state.

Leveraging this ADS concept, ICMS can induce measurable neurofunctional changes between recording and target areas. In healthy primates and rodents, precisely controlling the delay between the trigger spike and the target-site ICMS to 5 to 50 ms significantly induces a directional shift of M1 motor representations from the recording area toward the stimulation area [15] (Fig. 6B). Furthermore, this paradigm significantly reduces the threshold for evoking tactile perception in S1 [75] and enhances functional connectivity between the recording and target regions [76,115]. These phenomena operate independently of the physical distance between the recording and target sites [15,78] and are broadly consistent with the classical STDP time window [107,116]. In contrast, long-delay stimulation exceeding 50 ms [15,76] or continuous open-loop paired stimulation uncoupled from spontaneous activity [75,78,115] fails to induce stable or persistent STDP-like connectivity enhancements in vivo. Together, these comparisons indicate that timing contingency is a key determinant of ICMS-induced plasticity, and artificially coupling presynaptic neuronal firing with ICMS-induced postsynaptic depolarization provides a possible method for neural connectivity remodeling. However, most demonstrations remain tied to selected cortical targets, animal models, and short observation windows, so their scalability and relevance to durable therapeutic remodeling require further validation. They should not yet be interpreted as equivalent to natural experience-dependent sensory or motor plasticity [8].

In TBI models, ICMS also demonstrates potential for supporting circuit-level compensation after injury. For example, in rats with M1 lesions, researchers developed a closed-loop neural prosthesis for functional recovery by bridging spike activity in the rostral forelimb area (RFA) with ICMS in S1, effectively enhancing the functional connectivity between these 2 regions with a 7.5 to 10 ms delay. This paradigm significantly enhanced intercortical input–output coupling and increased stimulus-spike synchronization. At the same time, fine motor function of the rats’ forelimb recovered rapidly within 14 days and significantly outperformed open-loop stimulation and no-stimulation controls [77,117] (Fig. 6C). The researchers suggested that this behavioral benefit may arise from ICMS-induced enhancement of neural circuit connectivity. Chronic in vivo monitoring further confirmed that ADS can induce local up-regulation of synaptophysin around the electrode, providing anatomical support for enhanced synaptic connectivity induced by ICMS [118]. Additionally, multiple studies demonstrate that ADS-patterned ICMS rapidly modulates neuronal excitability [119–121]. Taken together, these findings support a repair-oriented interpretation in which temporally contingent ICMS first reshapes excitability and synchrony and may then promote pathway-level coupling. Notably, the functional neural connectivity sculpted by ICMS is not permanent. It typically persists robustly for a period before slowly returning to baseline over approximately 1 week [15,75–77]. This transient nature is primarily governed by the influence of homeostatic plasticity, which introduces another dimension of ICMS-mediated plasticity modulation and will be discussed in the following section. Overall, ICMS may help restore damaged functional connectivity, but current evidence cannot yet fully distinguish this effect from physiological functional compensation; more refined pathway-level monitoring and causal validation will be needed to address this question. Representative parameters for the ICMS plasticity-induction paradigms discussed here are summarized in Table 4.

**Table 4.** Representative stimulation parameters and electrode configurations in ICMS-based plasticity modulation studies

Study	Species	Target area	Electrode	Amplitude	Frequency	Pulse width	Interphase interval	Train duration	Experimental duration	Main findings
[15]	Macaque monkey (*N* = 2)	M1	Microwire	25–80 μA	ADS (~9–19 Hz)	200 μs per phase	NR	Single-pulse stimulation	1–4 d	M1 ADS induced lasting motor-output remapping
[76]	Rat (*N* = 3)	Forelimb sensorimotor cortex	Microwire	30 μA	ADS (≤10 Hz)	200 μs per phase	NR	Single-pulse stimulation	40–72 h	Short-delay ADS reshaped functional connectivity
[75]	Rat (*N* = 5)	Forelimb sensorimotor cortex	Microwire	20 μA	1,000 Hz	200 μs per phase	NR	Approximately 6 ms	48–72 h	Paired stimulation lowered ICMS detection thresholds
[115]	Macaque monkey (*N* = 2)	S1	Utah	50 μA	ADS/random stimulation (<10 Hz)	200 μs per phase	NR	2-pulse stimulation, ~2.8 ms	0.5 h	S1 spike-triggered stimulation enhanced tactile plasticity
[77]	Rat (*N* = 6)	S1; RFA (recording)	Michigan	60 μA	ADS (~8 Hz)	192 μs per phase	NR	Single-pulse stimulation	≤21 d	ADS improved reaching and RFA-S1 connectivity
[78]	Macaque monkey (*N* = 2)	S1/M1	Microwire	80–330 μA	300–330 Hz	NR	NR	6–6.7 ms	1–3 h	Paired stimulation induced STDP-like site strengthening
[117]	Rat (*N* = 11)	S1FL/S1BF; RFA (recording)	Michigan	60 μA	ADS (~7 Hz)	200 μs per phase	NR	Single-pulse stimulation	3 h	ADS enhanced distant coupling more than random stimulation
[118]	Rat (*N* = 5)	S1; RFA (recording)	Michigan	60 μA	ADS (~7 Hz)	200 μs per phase	NR	Single-pulse stimulation	21 d	Chronic ADS modulated RFA activity and S1 synaptic markers
[121]	Rat (*N* = 6)	S1; RFA (recording)	Michigan	60 μA	ADS (≤35 Hz)	200 μs per phase	NR	Single-pulse stimulation	1 h	Postinjury ADS increased firing and synchronized bursts

NR, not reported; RFA, rostral forelimb area; S1BF, S1 barrel-field region; S1FL, S1 forelimb region; h, hours, d, days

Achieving chronic ICMS modulation in freely behaving animals requires uninterrupted neural monitoring coupled with conditional stimulation delivery. Various integrated stimulation devices, including the Neurochip series [122–124], NeuroRighter [125,126], the wireless artifact-free neuromodulation device (WAND) [127], and the smart neurostimulation system (SNS) [128], have provided a technical basis for low-latency triggering, real-time artifact control, and programmable precise ICMS delivery; their technical parameters are summarized in Table 5. Recently, wireless distributed neuromodulation systems based on the “neurograin” concept [129] have emerged and may extend complex ICMS paradigms toward the whole-brain level [130]. Looking forward, miniaturized, low-power closed-loop devices integrated with on-chip machine learning will provide a reliable hardware basis for chronic ICMS-induced neural plasticity and for testing ADS-like plasticity rules, but their capacity for connectivity modulation should be jointly validated through long-term behavioral outcomes and safety assessment while considering activity-specific biomarkers, delay parameters, stimulation patterns, and safety boundaries, so that useful Hebbian strengthening, maladaptive synchronization, and compensatory downscaling can be distinguished [128,131,132].

**Table 5.** Comparison of technical parameters of closed-loop neuromodulation devices relevant to activity-dependent ICMS

Feature	Neurochip [15,122]	NeuroRighter [125,126]	Neurochip-2 [123]	WAND [127]	Neurochip-3 [124]	SNS [128]
Dimensions	44 mm × 20 mm × 15 mm	15.2 cm × 8.9 cm (not head-fixed)	55 mm × 35 mm× 20 mm	36 mm × 33 mm × 15 mm	56 mm × 40 mm × 50 mm	NR (coin-sized cranial IPG)
Wireless link	Infrared	NR (tethered)	Infrared	Nordic BLE	Infrared	Inductive IPG-EP link; EP USB/BLE
Recording channels	1 unipolar, 2 bipolar	16–64	3	128	16 bipolar/32 unipolar	60
Sample rate	11.7/2 kS·s^−1^	25 kS·s^−1^	2.6/5.3/10.5 kS·s^−1^	1 kS·s^−1^	5/10/20 kS·s^−1^	0.5 kS·s^−1^
ADC resolution	8 bits	16 bits	8 bits	15 bits	16 bits	NR
Stimulation channels	1	Up to 64	3	128	6	60
Maximum current	100 μA	NR	10–200 μA; up to 5 mA	5 mA	10 mA	5 mA
Biomarker	Action potential	Action potential	Action potential/spectral power	Spectral power	Action potential/spectral power	Spectral power
Closed-loop control	Event detection	Event detection	Event detection/feature thresholding	Feature thresholding	Event detection/feature thresholding	Spectral-feature classification
Artifact cancellation	No active cancellation	SALPA-compatible	Transient gain reduction	Linear interpolation	Fast-settle/blanking window	Poststimulation analysis window
Operation time	≤60 h	NR	≤48 h	~11.3 h	≤24 h	~8 h
Processing platform	On-board PSoC	Host PC/API	On-board PSoC	On-board FPGA/MCU	On-board MCU/FPGA	Implanted processor
Closed-loop timing	0.5 ms example	7.1–46.9 ms	5 ms example	8–1,024 ms	<50 ms supported	<25 ms
Demonstrated application	Cortical plasticity	Network control	Chronic recording/stimulation	Movement modulation	Circuit bridging	Spectral power modulation
Animal model	Nonhuman primate	Rodent/culture	Nonhuman primate	Nonhuman primate	Rodent/nonhuman primate	Ovine

NR, not reported; ADC, analog-to-digital converter; BLE, Bluetooth low energy; EP, external processor; FPGA, field-programmable gate array; IPG, implantable pulse generator; MCU, microcontroller unit; PSoC, programmable system-on-chip; SALPA, subtraction of artifacts by local polynomial approximation; WAND, wireless artifact-free neuromodulation device; SNS, smart neurostimulation system

### Modulating homeostatic plasticity for pathological network intervention

Homeostatic plasticity refers to a negative-feedback plasticity mechanism by which neurons and neural circuits counteract activity perturbations caused by learning, development, or injury by regulating synaptic strength, intrinsic excitability, and network excitation/inhibition (E/I) balance, thereby maintaining their activity within a stable functional range [133] (Fig. 5C). This concept originated from the “sliding threshold” in the Bienenstock–Cooper–Munro theory and later developed into the theories of metaplasticity and synaptic scaling, emphasizing how prior neuronal activity regulates subsequent synaptic changes to restrain excessive Hebbian positive-feedback loops in the form of LTP or LTD [134–136]. Consequently, cortical changes induced by tetanic high-frequency ICMS are generally difficult to sustain [75,78]. In contrast, sparse stimulation patterns triggered by spontaneous neural activity (such as spikes) more closely resemble physiological firing, making them less likely to trigger homeostatic down-regulation and potentially induce more robust and persistent remodeling of neural connectivity [15,77,118]. These phenomena indirectly suggest that stimulation paradigms consistent with physiological activity patterns may be more favorable for balancing Hebbian reinforcement with homeostatic regulation.

In diseases characterized by abnormal neural discharge, homeostatic plasticity can lead to an overcompensation of intrinsic excitability, thereby propelling disease progression [137]. ICMS may interfere with maladaptive coupling in these circuits, offering a potential strategy to modulate pathological homeostatic imbalances in such conditions. For instance, in focal epilepsy, the failure of inhibitory homeostatic mechanisms within the epileptogenic zone has been considered to increase α-amino-3-hydroxy-5-methyl-4-isoxazolepropionic acid receptor density in lesioned areas, thereby leading to the strengthening of pathological excitatory synaptic connections, suggesting that abnormal homeostatic plasticity involves both synaptic efficacy and network-level regulation [138]. In addition to directly interrupting epileptiform activity in the cortex [139], ICMS can also precisely modulate abnormal homeostatic plasticity through activity-dependent closed-loop paradigms. Interictal epileptiform discharges (IEDs) represent a meaningful neural activity target; under pathological conditions, they often form cross-regional abnormal coupling with normal brain oscillations (such as sleep spindles), which can, through chronic plasticity mechanisms, continuously expand the epileptic network and impair cognitive function [140–142]. Modulating interictal patterns may gradually reshape neural networks, support physiological brain function, and suppress epileptic activity, as preliminarily shown in cortical surface stimulation studies [143]. Previous studies have shown that, in a rat model of hippocampal kindling, the real-time detection of hippocampal IEDs followed by the application of Gaussian-waveform ICMS to the medial prefrontal cortex (mPFC) with a millisecond-level delay prevented pathological coupling between the 2 regions. This intervention could effectively delay the network-wide propagation of the epileptic focus and improve cognitive memory function [142,144]. These findings support the mechanistic feasibility of closed-loop ICMS for intervening in epilepsy-related pathological network coupling, but the current evidence still comes mainly from animal experiments and depends on selected biomarkers, stimulation targets, and timing rules. Therefore, compared with more macroscopic or clinically mature neuromodulation therapies such as vagus nerve stimulation (VNS), DBS, and responsive neurostimulation (RNS), ICMS is currently better defined as a cortical-circuit-level proof of concept [145].

Similarly, Parkinson’s disease (PD) is associated with chronic abnormalities in cortical homeostatic plasticity. Progressive dopamine depletion disrupts plasticity in the motor cortex and cortico-striatal circuits [146], leading to motor deficits. Levodopa and other dopaminergic therapies may partially restore this balance but may also induce aberrant plasticity [147]. This dual effect suggests that more precise and controllable plasticity-modulating approaches are needed to compensate for the limitations of dopaminergic therapy alone [148]. Current PD-related studies primarily use ICMS as a high-resolution functional detection tool to assess motor cortical excitability, motor representations, and cortico-basal ganglia loop changes in 6-hydroxydopamine or 1-methyl-4-phenyl-1,2,3,6-tetrahydropyridine models [149,150]. In addition, studies in PD models indicate that ICMS can suppress pathological electrical activity in mouse brain slices and transiently improve motor capacity when applied to rat M1 [151,152]. Meanwhile, theta-burst patterned cortical stimulation can modulate motor cortical excitability in patients with PD, induce LTP/LTD-like changes, and improve motor performance [153], supporting the broader potential of plasticity-based cortical modulation. Overall, current PD-related ICMS studies still lack clinical validation and evidence of durable functional benefit; ICMS is therefore better understood as a high-resolution experimental tool for probing and modulating cortical microcircuits [9,154]. With further integration of closed-loop paradigms, ICMS may expand its role in PD through more precise plasticity modulation [155].

In summary, ICMS has the potential to interfere with abnormal homeostatic plasticity, but its translational relevance should be interpreted in the context of established clinical neuromodulation therapies. Among invasive neuromodulation technologies, DBS is relatively similar to ICMS in intervention form but typically uses larger electrodes to deliver milliampere-level currents to deep nuclei such as the subthalamic nucleus, recruiting heterogeneous neuronal populations and fiber tracts within a several-millimeter region [9,156]. By contrast, ICMS delivers microampere-level pulses to cortical microcircuits and recruits neural elements over a range of tens of micrometers, making it better suited for targeting plasticity-related nodes within local cortical circuits. Recent studies further indicate that ICMS recruits excitatory and inhibitory neurons in an activity-state-dependent manner and can induce postactivation neural response depression, suggesting that patterned ICMS may influence long-range plastic reorganization by regulating local E/I balance [10,157]. In the future, closed-loop or specifically patterned ICMS may provide a high-precision cortical-level complementary strategy for selected disorders characterized by abnormal neural discharge. Before clinical translation, however, reliable biomarkers, reproducible parameters, implantation safety, and durable functional benefits require systematic validation [158,159].

Although ICMS-based electrical neuromodulation shows important potential in reshaping existing circuits and intervening in abnormal plasticity, its efficacy is fundamentally constrained by the long-term stability of the electrode interface and the requirement of intact neural substrates. To address these physical and biological limitations, research has begun to explore whether BNIs can improve electrode–tissue interface stability and signal transmission while extending the potential applications of ICMS in injury repair. This is the emerging frontier we explore next.

## BNIs for ICMS Applications

In chronic ICMS applications, BNIs can be understood as experimental strategies for interface stabilization, living signal bridging, and tissue repair. Whether rigid or flexible, MEAs remain constrained in long-term applications by the mismatch between device materials and brain tissue, which drives the need to integrate living biological components into neuroelectronic systems [43]. Compared with conventional electrodes that activate neurons through direct physical stimulation, BNIs introduce living neural components, creating possibilities for biointegration, synaptic bridging, and regenerative modulation, and may help overcome the limitations of purely physical stimulation [46,48], thereby offering new possible solutions for chronic ICMS. Current strategies can be read as a problem-oriented continuum: one group aims to improve the tissue–device interface or provide biologically mediated signal bridging, whereas another group, based on biohybrid regenerative electronics, further integrates stem cells, progenitors, or brain organoids to explore graft maturation, integration, and tissue reconstruction [19]. We provide a preliminary summary of representative studies of cortical BNIs in Table 6.

**Table 6.** Summary of representative studies of biohybrid neural interfaces

Study	Biological component	Electrode structure	Experimental model	Limitations
[44]	Rat sciatic nerve segment; host cortical neurites	Hollow glass cone electrode	Rat S1	Tissue-ingrowth dependent; limited channels
[160]	Human host cortical neurites	Hollow neurotrophic glass cone	Human motor speech cortex	Complex implantation; limited scalability
[162]	E14 mouse cortical NSCs	Parylene probe with microwell for NSCs	Rat cortex	Chronic benefit not maintained
[164]	Adult mouse subependymal NPCs	Silicon probe seeded with NPCs	Rat cortex and in vitro testing	Chronic performance not tested
[163]	E18 rat hippocampal neurons; rat astrocytes	Fibrin-coated microelectrode	Rat cortex and in vitro testing	Living-cell contribution unclear
[184]	E18 rat cortical neural cells	Silk film with patterned gold electrodes	Mouse cortex and in vitro testing	In vivo fully cell-loaded electrode function not tested
[16]	Rat neuroprogenitors/glial cells	Platinum electrode with conductive hydrogel	In vitro testing	In vitro only; integration untested
[161]	Human host neurites	Hollow neurotrophic glass cone	Human motor speech cortex	Single case; low throughput
[185]	Human hESC-derived NPCs	PPy/PDMS conductive polymer	Rat cortical stroke model	Long-term integration unproven
[187]	Human hiPSC-derived cortical organoids	Transparent graphene MEA	Mouse cortex	Stimulation/repair not tested
[165]	E18 mouse cortical neurons	4 × 4 biohybrid transition array	Rat cadaver cortex and in vitro testing	Proof of concept only
[188]	Human hESC-derived brain organoids	Organoid-mediated OBCI	Mouse S1 injury model	Long-term safety unvalidated
[166]	E18 rat neural spheroids/axon bundles	Stretchable array with spheroid wells	Mouse cortex and in vitro testing	In vivo synapses not achieved
[186]	E13.5 mouse NSCs	Living artificial dura seeded with NSCs	Mouse cortical TBI model	Integration and safety unvalidated

E, embryonic day; hESC, human embryonic stem cell; hiPSC, human induced pluripotent stem cell; NPCs, neural progenitor cells; NSCs, neural stem cells; OBCI/OBCIs, organoid–brain–computer interface(s)

### BNI strategies for interface integration

For BNIs, the earliest and still instructive question is whether the host tissue can be induced to couple with an implanted structure more actively than it does with a conventional inert electrode. A classic strategy was the cone electrode, which used a sciatic nerve graft to guide host cortical neurites into the tip of a glass cone electrode and could support stable recordings for months [44]. Subsequent work replaced the sciatic nerve graft with neurotrophic factors and refined this strategy into the neurotrophic electrode, which supported approximately a decade of stable recording from human motor speech cortex, with histological evidence showing myelinated neural filaments inside the electrode and no obvious gliotic encapsulation [160,161]. Although these electrodes remain complex to implant, low in channel count, limited in scalability, and mainly supported by structural and recording evidence, they demonstrate the feasibility of incorporating living tissue into electrode structures and provide an early conceptual basis for the use of BNIs in chronic ICMS and bidirectional BCIs.

Another strategy uses cell-seeded electrode interfaces, in which living cells are directly loaded onto probe surfaces to mitigate electrode-implantation-induced FBR. Representative strategies include direct seeding of NSCs, neural progenitor cells (NPCs), and other neural cells on polymer or rigid probes through hydrogel- or protein-modified coatings. In selected experiments, these methods improved early peri-electrode neuronal density, cell adhesion, or electrochemical performance [162–164]. Compared with cone and neurotrophic electrodes, these designs more directly target the tissue–device interface, but their effects remain time-dependent. For example, early benefits of probes loaded with NSCs were not maintained and even reversed by 6 weeks, whereas other studies were mainly evaluated within implantation windows shorter than 1 month. Therefore, their long-term development still requires further resolution of cell survival and coating stability so that durable biological buffering can be maintained under chronic ICMS conditions.

Further emerging strategies move beyond passive interface modification by using engineered microstructures to guide cell growth and living signal bridging. The original “living electrode” proposed a layered hydrogel structure that combines conductivity with cell support [16]. Subsequently, one prototype study integrated neuron-loaded microwells, gelatin methacrylate-filled polymer microneedles, and axon-guiding microchannels into a biohybrid transition MEA, achieving directed axonal growth and synaptic connection formation, although functional signal transmission has not yet been validated in vivo [165]. Spheroid-based interfaces further brought this idea in vivo by combining neural spheroids, stretchable electrode arrays, and microfluidic channels; they supported axon-mediated signal conduction within 5 days after implantation, but subsequent axonal degeneration and neuroinflammation were observed [166].

These studies mark a shift in BNIs from passive compatibility toward living signal transfer, suggesting a possible route for maintaining chronic ICMS interfaces and supporting biologically coupled signal transmission. However, current evidence still comes mainly from in vitro or short-term animal validation. For such systems to develop into long-term stable functional interfaces, cell viability, immune compatibility, and chronic ICMS tolerance must be demonstrated together.

### BNIs based on biohybrid regenerative electronics

ICMS can induce synaptic plasticity within cortical neural networks. However, when TBI, stroke, or other causes result in the loss of neural tissue, neuromodulation loses its viable targets. Relying solely on the plasticity of surrounding intact tissues is often insufficient to achieve complete functional recovery. In this context, BNIs based on biohybrid regenerative electronics differ from the interface-integration or signal-bridging strategies discussed above. By combining cell transplantation with electrode interfaces, they may further extend ICMS applications toward damaged-network repair, host–graft connections, and functional reconstruction [46,48]. This direction is supported by the convergence of 2 technical approaches: in vitro MEA platforms [167,168], which enable long-term recording and stimulation of NSCs, NPCs, and brain organoids, and in vivo brain interfaces, which provide chronic recording, stimulation, and potential closed-loop modulation after graft implantation [45,47]. This in vitro-to-in vivo continuity not only supports the development of BNIs based on biohybrid regenerative electronics but also imposes higher requirements for graft survival, integration, and control over graft fate.

#### Electrical regulation of stem cell and organoid fates

**Fig. 7.**
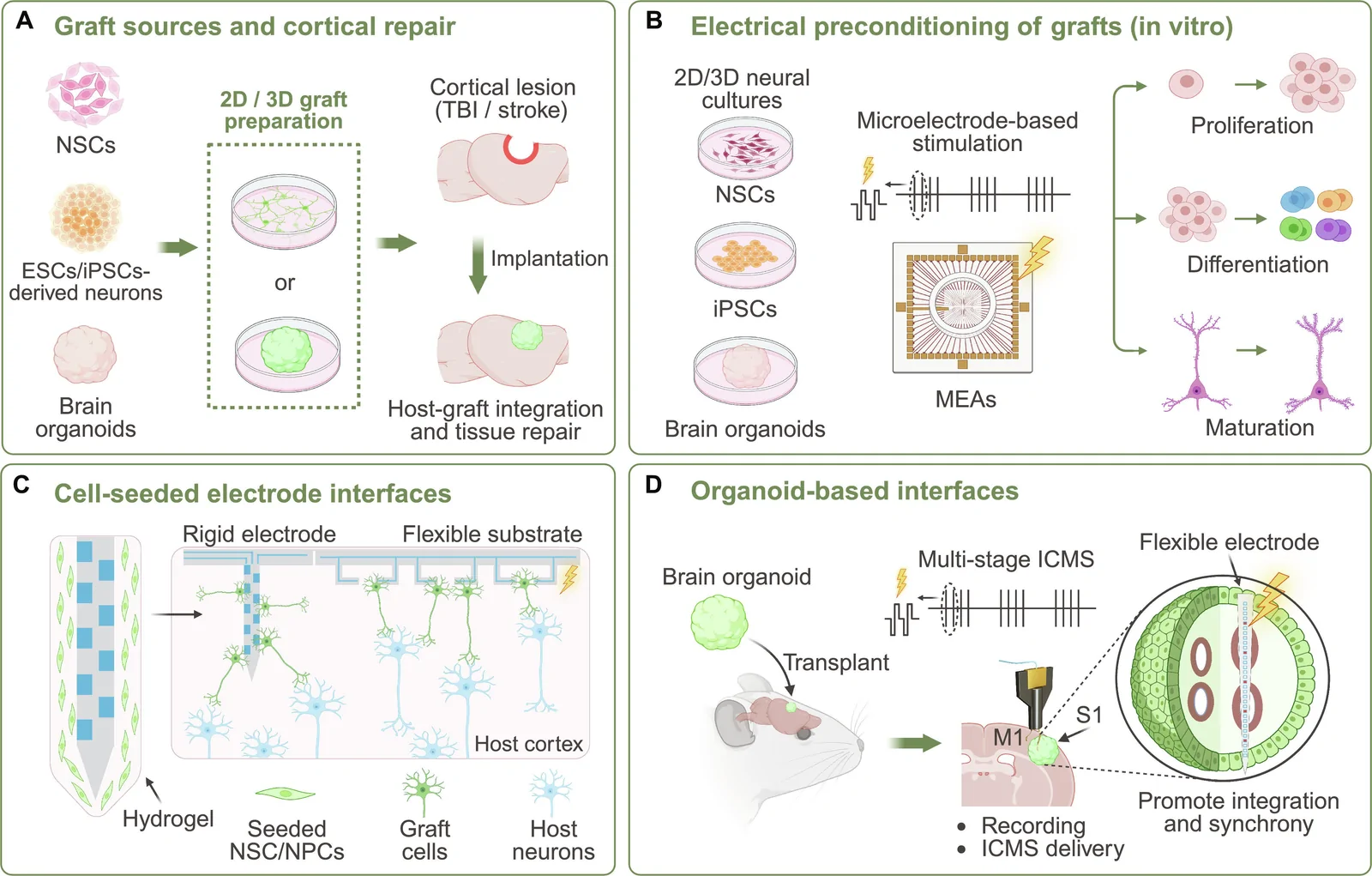
In vitro graft modulation and in vivo integration in BNIs. (A) NSCs, ESC- or iPSC-derived neurons, and brain organoids can be delivered as 2D or 3D grafts into cortical lesions to support host–graft integration and tissue repair. (B) Microelectrode-based electrical stimulation can modulate the proliferation, differentiation, and maturation of 2D or 3D neural cultures and may be used for graft preconditioning. (C) Cell-seeded electrode interfaces use hydrogel- or surface-modified rigid electrodes or flexible substrates primarily to support peri-electrode integration and mitigate FBR, with selected flexible systems also enabling electrical modulation of transplanted NPCs or NSCs. (D) Brain organoids are transplanted into the mouse S1 and coupled with flexible electrode arrays to enable recording and ICMS delivery during selected posttransplantation windows. This paradigm may support host–graft synchrony and integration.

In the damaged cortex, transplanted NSCs [169], neurons derived from embryonic stem cells (ESCs) or induced pluripotent stem cells (iPSCs) [170,171], and brain organoids [172] can fill lesion cavities, chronically integrate with the host tissue, and actively participate in neural network activity, representing a potential neurorestorative strategy (Fig. 7A). However, whether in cell transplantation alone or biohybrid electronics-based cell implantation, grafts require posttransplantation guidance, and the growth of implanted cells must be controlled to support integration, reduce cell loss, and lower the risk of abnormal growth [173,174], highlighting the importance of ICMS-based regulation of stem cells transplanted in vivo.

In vitro studies demonstrate that microelectrode-based electrical stimulation can modulate stem cell fate [174], effectively inducing the proliferation [175], differentiation, and maturation [176] of 2D NSC or iPSC cultures. As these cultures evolve from 2D to 3D structures, the advantages of electrical modulation become even more pronounced. 3D brain organoids can closely simulate neurogenesis, cortical stratification, cell diversity, and neural circuits of the human brain [177]. By utilizing 3D microelectrode cantilevers that penetrate into the tissue interior [178,179], ICMS-style electrical pulses not only promote organoid maturation and differentiation [180] but also support the modeling of neurological diseases using these organoids as platforms [181]. Furthermore, preconditioning with microelectrode-based electrical stimulation can also improve the viability of 2D or 3D cultures as transplant donors and enhance the quality of posttransplant integration [182,183]. These findings support the view that in vivo ICMS may regulate the proliferation, differentiation, and maturation of stem cells, progenitors, or brain organoids, thereby potentially compensating for low graft survival and nondirectional circuit integration [173] and providing preliminary support for constructing BNIs based on biohybrid regenerative electronics [47] (Fig. 7B).

#### In vivo integration and regenerative circuit repair

In biohybrid regenerative electronics, combining NPCs or NSCs with surface-modified flexible interfaces and electrical modulation may extend the role of cell-seeded electrode interfaces beyond local interface support toward host–graft coupling (Fig. 7C). An early study provided initial support for this interface strategy: rat cortical neurons seeded on silk films patterned with gold electrodes were associated with a limited inflammatory response after implantation in the mouse cortex [184]. In a rat model of M1 stroke, PPy-modified PDMS flexible microchambers enabled delivery and electrical modulation of human NPCs, significantly up-regulated stanniocalcin-2 expression in transplanted NPCs, accelerated sensorimotor recovery, and promoted endogenous stem cell proliferation [185]. In another study, a living artificial dura mater constructed by seeding NSCs onto a magnetically driven Au-nanostrip patch promoted the differentiation of transplanted NSCs and activated local neuroprotective A2 astrocytes, thereby remodeling the post-TBI immune-inflammatory microenvironment [186]. These studies suggest that ICMS-based BNIs can move beyond passive interface modification by regulating transplanted cell fate and gradually approach active repair. However, long-term stimulation/recording control and stable graft–host circuit integration have not yet been achieved, and further development toward closed-loop modulation systems remains necessary.

Beyond preseeding cells onto electrode surfaces, an alternative effective strategy involves allowing the living graft to initially colonize the host tissue in vivo before applying modulation via MEAs. Brain organoid transplantation provides a more complex 3D substrate for BNIs: organoids can integrate with injured host cortex, form bidirectional synaptic connections, participate in host neural activity, and be monitored over time using MEAs [187]. On this basis, recent work on organoid–brain–computer interfaces (OBCIs) has advanced by introducing active ICMS modulation. By transplanting brain organoids into the S1 cortical lesion area of mice and implanting flexible electrode probes, researchers constructed an OBCI with both signal acquisition and ICMS delivery functions (Fig. 7D). Applying 10 days of ICMS at 30 and 60 days after transplantation could promote organoid maturation and integration while significantly enhancing electrophysiological coupling between the organoids and the host and improving mouse von Frey nociceptive behavioral performance, suggesting that ICMS could promote functional integration between the organoids and host cortex [188]. By establishing a bidirectional channel for stimulation and recording, this paradigm provides a novel perspective on utilizing BNIs to integrate with the host network for brain-injury repair. Compared with 2D stem-cell grafts, organoid-based BNIs have the advantages of being 3D and amenable to monitoring, but they still require longer in vivo validation of posttransplantation survival, vascularization, projection specificity, and electrophysiological function, while tumorigenicity should be evaluated as an independent safety dimension [187,188].

In summary, ICMS-oriented BNIs combine the high spatiotemporal resolution of ICMS with the bioactivity and regenerative capacity of biological tissues, and may become an emerging framework for chronic neural interfaces and graft–host network modulation. However, current evidence should still be interpreted cautiously: existing studies differ in cell source, interface structure, implantation site, and final outcomes. Their near-term value is not that they already solve the limitations of chronic ICMS, but that they provide testable routes along the same continuum: improving the interface, bridging signals, and guiding repair-oriented host–graft coupling. Future work should compare and evaluate these strategies in terms of cell fate, immune response, signal fidelity, stimulation controllability, and ethical governance, and should establish evaluation boundaries for BNIs with different research goals [19,43,46–48].

## Challenges and Future Perspectives

Taken together, the future translation of ICMS can be understood within the integrative framework established in this review: hardware interfaces influence chronic usability, stimulation parameters delimit neural recruitment and safety boundaries, artificial-perception encoding shapes sensory-feedback quality and task support, plasticity modulation determines whether ICMS can extend into a circuit-intervention tool, and BNIs expand its potential for in vivo applications. Accordingly, future challenges mainly concentrate on 5 aspects: chronic reliability, mechanistic encoding and calibration, translational positioning, safety and ethics, and governance of BNIs.

**1. Chronic interface reliability and hardware scalability**

Currently, hardware-interface reliability remains a major bottleneck for chronic ICMS applications. Long-term Utah-array studies in humans and nonhuman primates support the feasibility of chronic ICMS, yet they also reveal heterogeneity in recording quality, perceptual thresholds, electrode stability, and tissue responses [30,31,66,93]. Therefore, ICMS hardware development should not be understood simply as replacing rigid electrodes with flexible electrodes. Chronic reliability and scalability should instead be treated as core engineering goals, with mature and emerging routes integrated in stages. Current studies support this staged view. Flexible electrodes are moving toward nanoscale and ultraflexible designs with advanced manufacturing technologies, but their long-term insulation integrity and electrochemical stability in complex in vivo environments still require rigorous validation [19]. Conventional rigid electrodes remain important for near-term clinical translation, and their chronic use can be optimized at the levels of interface design, stimulation delivery, and system packaging: bioactive surface modification may improve local tissue compatibility [32,33], multielectrode stimulation may reduce the burden on individual contacts [61,66], and wireless or fully implantable packaging may help support long-term use [127–129,189]. In the future, the hardware foundation for ICMS should be able to maintain stable performance over years.

**2. Mechanistic encoding and calibration for artificial perception**

ICMS can now evoke distinguishable tactile and visual artificial percepts. With training, these percepts or stimulation patterns can also serve as task-related cues, but their translational value still depends on whether such artificial inputs can remain stable, interpretable, and usable over the long term. Current work has produced 2 complementary strategies. Spatially, multielectrode synchronous or spatiotemporally patterned stimulation can adjust activation range, distribute channel burden, and generate patterned percepts. Temporally, biomimetic dynamic amplitude or frequency modulation can improve tactile naturalness and reduce perceptual fading caused by sustained activation [62–64,190–192]. However, perceptual encoding and stimulation-recruitment mechanisms remain insufficiently defined. Future studies should clarify how stimulation amplitude, frequency, pulse width, pulse shape, multielectrode recruitment, and cortical layers affect perceptual quality, safety boundaries, and long-term stability, while integrating electrode design, encoding algorithms, subjective reports, and closed-loop calibration to promote more scalable sensory neuroprostheses. At the same time, a bidirectional translational framework could align human perceptual performance, animal mechanistic recordings, and computational models, so that perceptual quality, threshold drift, or perceptual fading observed in humans can become mechanistic clues for animal experiments, cross-species parameter calibration, and human ICMS parameter optimization [8,60].

**3. Translational positioning of ICMS within neuromodulation**

As ICMS moves from transient percept evocation toward plasticity modulation, it should be positioned within the broader neuromodulation landscape. DBS, VNS, RNS, TMS, and tDCS already occupy distinct roles across clinical and experimental settings, with different trade-offs in invasiveness, stimulation scale, spatial specificity, safety, and maturity [4–6,9,145]. In this context, ICMS primarily acts on local cortical microcircuits and is better positioned as a complement rather than a replacement for existing clinical neuromodulation methods. More feasible translational directions include local microcircuit probing, cortical information input, and closed-loop plasticity induction. ICMS may also provide a mechanistic reference for larger-scale neuromodulation such as DBS [60]. Current closed-loop and distributed stimulation systems already reflect this trend by linking recording, stimulation, timing rules, and behavioral or network outcomes [15,76,77,127–129,142,144,189,193]. For diseases characterized by homeostatic imbalances, such as epilepsy and PD, the effects of ICMS should be evaluated in terms of target scale, threshold reproducibility, decoding reliability, functional benefit, surgical risk, regulatory feasibility, and patient variability. Future studies should also include sham or active-stimulation controls, longer observation windows, and cross-species parameter mapping to determine whether ICMS can induce durable and beneficial plastic changes, intervene in disease progression, and avoid disrupting physiological circuit function [194,195].

**4. Safety and ethical constraints of ICMS**

Building on the interface reliability, stimulation strategy, and chronic-use constraints discussed above, the safety and ethical limitations of clinical ICMS require more comprehensive consideration. As an invasive technology, electrode implantation involves surgical risks, including hemorrhage, infection, and acute tissue injury, and long-term use also involves chronic tissue responses, threshold drift, and electrode failure [8,159]. During application, ICMS may also affect surrounding glial cells and pericytes, potentially supporting long-term use by modulating local vascular function and tissue responses, but this direction still lacks chronic human evidence [157,196]. Therefore, safety evaluation should run throughout ICMS application rather than being handled only at the final regulatory stage. Technical efforts such as standardized array implantation roadmaps and robot-assisted array implantation are being developed to reduce placement variability and procedural burden [3,36]. At the same time, because ICMS directly delivers signals to the brain as an information-input strategy in invasive BCIs, its applications should also consider informed consent, patient psychology, long-term device management, and posttrial responsibility [197], and should establish comprehensive and stringent standards for identity authentication, information encryption, and system protection to safeguard neural-data privacy [198].

**5. Controllability and governance of BNIs**

BNIs should be developed into controllable and governable platforms rather than remain conceptually attractive extensions of electrode technology. The development of BNIs reflects multiple technical routes: cell-seeded coating strategies aim to improve interface integration, axon-guidance strategies aim to support living signal bridging, and stem-cell- or organoid-integrated strategies aim to guide regenerative coupling [16,19,43,45–47,162,163,166,188]. Feasible supporting technologies include microfluidic control of the local environment, on-site oxygen generation and immunoprotection, wireless multifunctional living interfaces, vascularized organoids, and 3D flexible electrodes for organoid electrophysiology [199–202]. At the same time, more exploratory cell-based BCI concepts have started to appear in industry-oriented proposals. For example, a Neuralink-related patent proposes transplanting genetically modified neurons above cortical layer 1 (L1) to form an artificial cortical layer zero (L0), potentially enabling bidirectional communication through thin-film microelectrodes or related formats, making this layer an input/output layer for BCIs [203]. Furthermore, stringent safety evaluation systems must be established, including assessment of long-term survival of living components, immune compatibility, standardized manufacturing, regulatory classification, and ethical acceptability, with the goal of ensuring the safe and controllable integration of living grafts with the host cortex while guaranteeing the long-term quality and reliability of stimulation [47,48].

Further maturation and translation of ICMS require systematic validation across hardware interfaces, stimulation encoding, closed-loop modulation, disease-specific applications, and biointegration. Future studies should combine cross-species experiments, long-term follow-up, standardized safety evaluation, and reproducible behavioral and network outcomes, with criteria tailored to microelectrode materials, stimulation parameters, perceptual encoding, closed-loop algorithms, and BNIs. These studies should assess long-term usability, mechanistic interpretability, functional or therapeutic benefit, and safety to define the applicable settings and translational value of each technical route and build a continuous evidence chain from experimental microstimulation to chronically usable BCI information interfaces and precision neuromodulation.

## Data Availability

No new data were created or analyzed in this study.

## References

[1] Robinson JT, Norman SL, Angle MR, Constandinou TG, Denison T, Donoghue JP, Field RM, Forsland A, Kouider S, Millán JD, et al. An application-based taxonomy for brain–computer interfaces. Nat Biomed Eng. 2025;9(6):789–791. 39715900 10.1038/s41551-024-01326-z

[2] Downey JE, Schone HR, Foldes ST, Greenspon C, Liu F, Verbaarschot C, Biro D, Satzer D, Moon CH, Coffman BA, et al. A roadmap for implanting electrode arrays to evoke tactile sensations through Intracortical stimulation. Hum Brain Mapp. 2024;45(18): Article e70118. 39720868 10.1002/hbm.70118 PMC11669040

[3] Ikegaya N, Mallela AN, Warnke PC, Kunigk NG, Liu F, Schone HR, Verbaarschot C, Hatsopoulos NG, Downey JE, Boninger ML, et al. A novel robot-assisted method for implanting intracortical sensorimotor devices for brain-computer interface studies: Principles, surgical techniques, and challenges. J Neurosurg. 2024;142(5):1280–1288. 39642366 10.3171/2024.7.JNS241296 PMC12445443

[4] Qi Z, Liu H, Jin F, Wang Y, Lu X, Liu L, Yang Z, Fan L, Song M, Zuo N, et al. A wearable repetitive transcranial magnetic stimulation device. Nat Commun. 2025;16(1):2731. 40108144 10.1038/s41467-025-58095-9 PMC11923099

[5] Fehring DJ, Yokoo S, Abe H, Buckley MJ, Miyamoto K, Jaberzadeh S, Yamamori T, Tanaka K, Rosa MG, Mansouri FA. Direct current stimulation modulates prefrontal cell activity and behaviour without inducing seizure-like firing. Brain. 2024;147(11):3751–3763. 39166526 10.1093/brain/awae273 PMC11531852

[6] Keser Z, Ikramuddin S, Shekhar S, Feng W. Neuromodulation for post-stroke motor recovery: A narrative review of invasive and non-invasive tools. Curr Neurol Neurosci Rep. 2023;23(12):893–906. 38015351 10.1007/s11910-023-01319-6

[7] Zhou J, Khateeb K, Yazdan-Shahmorad A. Early intervention with electrical stimulation reduces neural damage after stroke in non-human primates. Nat Commun. 2025;16(1):6701. 40691150 10.1038/s41467-025-61948-y PMC12280128

[8] Hughes C, Chen X, Grill W, Kozai TDY. Neural mechanisms underlying intracortical microstimulation for sensory restoration. Nat Biomed Eng. 2026;10(2):197–213. 41540147 10.1038/s41551-025-01583-6 PMC12947168

[9] Krauss JK, Lipsman N, Aziz T, Boutet A, Brown P, Chang JW, Davidson B, Grill WM, Hariz MI, Horn A, et al. Technology of deep brain stimulation: Current status and future directions. Nat Rev Neurol. 2021;17(2):75–87. 33244188 10.1038/s41582-020-00426-z PMC7116699

[10] Dadarlat MC, Sun YJ, Stryker MP. Activity-dependent recruitment of inhibition and excitation in the awake mammalian cortex during electrical stimulation. Neuron. 2024;112(5):821–834.e4. 38134920 10.1016/j.neuron.2023.11.022 PMC10949925

[11] Mazurek KA, Schieber MH. Injecting information into the mammalian cortex: Progress, challenges, and promise. Neuroscientist. 2021;27(2):129–142. 32648527 10.1177/1073858420936253 PMC7995349

[12] Penfield W, Boldrey E. Somatic motor and sensory representation in the cerebral cortex of man as studied by electrical stimulation. Brain. 1937;60(4):389–443.

[13] Flesher SN, Collinger JL, Foldes ST, Weiss JM, Downey JE, Tyler-Kabara EC, Bensmaia SJ, Schwartz AB, Boninger ML, Gaunt RA. Intracortical microstimulation of human somatosensory cortex. Sci Transl Med. 2016;8(361): Article 361ra141. 27738096 10.1126/scitranslmed.aaf8083

[14] Chen X, Wang F, Fernandez E, Roelfsema PR. Shape perception via a high-channel-count neuroprosthesis in monkey visual cortex. Science. 2020;370(6521):1191–1196. 33273097 10.1126/science.abd7435

[15] Jackson A, Mavoori J, Fetz EE. Long-term motor cortex plasticity induced by an electronic neural implant. Nature. 2006;444(7115):56–60. 17057705 10.1038/nature05226

[16] Goding J, Robles UA, Poole-Warren L, Lovell N, Martens P, Green R. A living electrode construct for incorporation of cells into bionic devices. MRS Commun. 2017;7(3):487–495.

[17] Xu L, Hu C, Huang Q, Jin K, Zhao P, Wang D, Hou W, Dong L, Hu S, Ma H. Trends and recent development of the microelectrode arrays (MEAs). Biosens Bioelectron. 2021;1(175): Article 112854. 10.1016/j.bios.2020.112854 33371989

[18] Siwakoti U, Jones SA, Kumbhare D, Cui XT, Castagnola E. Recent progress in flexible microelectrode arrays for combined electrophysiological and electrochemical sensing. Biosensors. 2025;15(2):100. 39997002 10.3390/bios15020100 PMC11853293

[19] Boufidis D, Garg R, Angelopoulos E, Cullen DK, Vitale F. Bio-inspired electronics: Soft, biohybrid, and “living” neural interfaces. Nat Commun. 2025;16(1):1861. 39984447 10.1038/s41467-025-57016-0 PMC11845577

[20] Yi D, Yao Y, Wang Y, Chen L. Manufacturing processes of implantable microelectrode Array for *in vivo* neural electrophysiological recordings and stimulation: A state-of-the-art review. J Micro Nanomanuf. 2022;10(4): Article 041001. 37860671 10.1115/1.4063179 PMC10583290

[21] Vafaiee M, Mohammadpour R, Vossoughi M, Asadian E, Janahmadi M, Sasanpour P. Carbon nanotube modified microelectrode array for neural interface. Front Bioeng Biotechnol. 2021;13(8): Article 582713. 10.3389/fbioe.2020.582713 PMC7839404 33520951

[22] Tanwar A, Gandhi HA, Kushwaha D, Bhattacharya J. A review on microelectrode array fabrication techniques and their applications. Mater Today Chem. 2022;26: Article 101153.

[23] Zhang S, Song Y, Wang M, Zhang Z, Fan X, Song X, Zhuang P, Yue F, Chan P, Cai X. A silicon based implantable microelectrode array for electrophysiological and dopamine recording from cortex to striatum in the non-human primate brain. Biosens Bioelectron. 2016;85:53–61. 27155116 10.1016/j.bios.2016.04.087

[24] Zhang S, Song Y, Lv S, Jing L, Wang M, Liu Y, Xu W, Jiao P, Zhang S, Wang M, et al. Electrode arrays for detecting and modulating deep brain neural information in primates: A review. Cyborg Bionic Syst. 2025;6: Article 0249. 40321898 10.34133/cbsystems.0249 PMC12046227

[25] Wise KD, Angell JB, Starr A. An integrated-circuit approach to extracellular microelectrodes. IEEE Trans Biomed Eng. 1970;31(3):238–247. 10.1109/tbme.1970.4502738 5431636

[26] Campbell PK, Jones KE, Huber RJ, Horch KW, Normann RA. A silicon-based, three-dimensional neural interface: Manufacturing processes for an intracortical electrode array. IEEE Trans Biomed Eng. 1991;38(8):758–768. 1937509 10.1109/10.83588

[27] Budday S, Nay R, de Rooij R, Steinmann P, Wyrobek T, Ovaert TC, Kuhl E. Mechanical properties of gray and white matter brain tissue by indentation. J Mech Behav Biomed Mater. 2015;46:318–330. 25819199 10.1016/j.jmbbm.2015.02.024 PMC4395547

[28] Yan Z, Gao W, Duan Y, Zhou H, Ni C, Huang L, Ye Z. Bidirectional mechanisms and emerging strategies for implantable bioelectronic interfaces. Bioact Mater. 2025;52:634–667. 40607118 10.1016/j.bioactmat.2025.06.014 PMC12221386

[29] Thielen B, Meng E. A comparison of insertion methods for surgical placement of penetrating neural interfaces. J Neural Eng. 2021;18(4): Article 041003. 10.1088/1741-2552/abf6f2 PMC8600966 33845469

[30] Colachis SC, Dunlap CF, Annetta NV, Tamrakar SM, Bockbrader MA, Friedenberg DA. Long-term intracortical microelectrode array performance in a human: A 5 year retrospective analysis. J Neural Eng. 2021;18(4): Article 0460d7. 10.1088/1741-2552/ac1add 34352736

[31] Chen X, Wang F, Kooijmans R, Klink PC, Boehler C, Asplund M, Roelfsema PR. Chronic stability of a neuroprosthesis comprising multiple adjacent Utah arrays in monkeys. J Neural Eng. 2023;20(3): Article 036039. 37386891 10.1088/1741-2552/ace07e PMC7617000

[32] Geramifard N, Khajehzadeh M, Dousti B, Abbott JR, Nguyen CK, Hernandez-Reynoso AG, Joshi-Imre A, Varner VD, Cogan SF. Flexible and extensible ribbon-cable interconnects for implantable electrical neural interfaces. ACS Appl Mater Interfaces. 2024;16(45):61621–61632. 39476818 10.1021/acsami.4c11773

[33] Yang M, Yang T, Deng H, Wang J, Ning S, Li X, Ren X, Su Y, Zang J, Li X, et al. Poly(5-nitroindole) thin film as conductive and adhesive interfacial layer for robust neural Interface. Adv Funct Mater. 2021;31(49):2105857.

[34] Wu X, Ye Y, Sun M, Mei Y, Ji B, Wang M, Song E. Recent progress of soft and bioactive materials in flexible bioelectronics. Cyborg Bionic Syst. 2025;6: Article 0192. 40302943 10.34133/cbsystems.0192 PMC12038164

[35] Chiang CH, Won SM, Orsborn AL, Yu KJ, Trumpis M, Bent B, Wang C, Xue Y, Min S, Woods V, et al. Development of a neural interface for high-definition, long-term recording in rodents and nonhuman primates. Sci Transl Med. 2020;12(538): Article eaay4682. 32269166 10.1126/scitranslmed.aay4682 PMC7478122

[36] Musk E. An integrated brain-machine interface platform with thousands of channels. J Med Internet Res. 2019;21(10): Article e16194. 31642810 10.2196/16194 PMC6914248

[37] Lycke R, Kim R, Zolotavin P, Montes J, Sun Y, Koszeghy A, Altun E, Noble B, Yin R, He F, et al. Low-threshold, high-resolution, chronically stable intracortical microstimulation by ultraflexible electrodes. Cell Rep. 2023;42(6): Article 112554. 37235473 10.1016/j.celrep.2023.112554 PMC10592461

[38] Liu J, Fu TM, Cheng Z, Hong G, Zhou T, Jin L, Duvvuri M, Jiang Z, Kruskal P, Xie C, et al. Syringe-injectable electronics. Nat Nanotechnol. 2015;10(7):629–636. 26053995 10.1038/nnano.2015.115 PMC4591029

[39] Zhang A, Mandeville ET, Xu L, Stary CM, Lo EH, Lieber CM. Ultraflexible endovascular probes for brain recording through micrometer-scale vasculature. Science. 2023;381(6655):306–312. 37471542 10.1126/science.adh3916 PMC11412271

[40] Abidian MR, Corey JM, Kipke DR, Martin DC. Conducting-polymer nanotubes improve electrical properties, mechanical adhesion, neural attachment, and neurite outgrowth of neural electrodes. Small. 2010;6(3):421–429. 20077424 10.1002/smll.200901868 PMC3045566

[41] Adewole DO, Serruya MD, Wolf JA, Cullen DK. Bioactive neuroelectronic interfaces. Front Neurosci. 2019;13:269. 30983957 10.3389/fnins.2019.00269 PMC6449725

[42] Tang X, Shen H, Zhao S, Li N, Liu J. Flexible brain–computer interfaces. Nat Electron. 2023;6(2):109–118.

[43] Rochford AE, Carnicer-Lombarte A, Curto VF, Malliaras GG, Barone DG. When bio meets technology: Biohybrid neural interfaces. Adv Mater. 2020;32(15):1903182. 10.1002/adma.201903182 31517403

[44] Kennedy PR. The cone electrode: A long-term electrode that records from neurites grown onto its recording surface. J Neurosci Methods. 1989;29(3):181–193. 2796391 10.1016/0165-0270(89)90142-8

[45] Yu YL, Muzaffar J, Philips V, Yang YJ, Barone DG, Malliaras GG, Bance M. Current developments and challenges in the field of biohybrid neural interfaces—A scoping review. Adv Electron Mater. 2025;11(19): Article e00259.

[46] Boulingre M, Portillo-Lara R, Green RA. Biohybrid neural interfaces: Improving the biological integration of neural implants. Chem Commun. 2023;59(100):14745–14758. 10.1039/d3cc05006h PMC10720954 37991846

[47] Hong M, Ye Y, Kim J, Kim JI, Rah JC, Tchoe Y. Integrating living biomaterials into neuroelectronic systems. Biomed Eng Lett. 2026;16(2):307–328. 41890249 10.1007/s13534-026-00557-0 PMC13013916

[48] Carnicer-Lombarte A, Malliaras GG, Barone DG. The future of biohybrid regenerative bioelectronics. Adv Mater. 2025;37(3):2408308. 39564751 10.1002/adma.202408308 PMC11756040

[49] Histed MH, Ni AM, Maunsell JHR. Insights into cortical mechanisms of behavior from microstimulation experiments. Prog Neurobiol. 2013;103:115–130. 22307059 10.1016/j.pneurobio.2012.01.006 PMC3535686

[50] Tehovnik EJ, Tolias AS, Sultan F, Slocum WM, Logothetis NK. Direct and indirect activation of cortical neurons by electrical microstimulation. J Neurophysiol. 2006;96(2):512–521. 16835359 10.1152/jn.00126.2006

[51] Butovas S, Schwarz C. Spatiotemporal effects of microstimulation in rat neocortex: A parametric study using multielectrode recordings. J Neurophysiol. 2003;90(5):3024–3039. 12878710 10.1152/jn.00245.2003

[52] Stoney SD, Thompson WD, Asanuma H. Excitation of pyramidal tract cells by intracortical microstimulation: Effective extent of stimulating current. J Neurophysiol. 1968;31(5):659–669. 5711137 10.1152/jn.1968.31.5.659

[53] Kumaravelu K, Sombeck J, Miller LE, Bensmaia SJ, Grill WM. Stoney vs Histed: Quantifying the spatial effects of intracortical microstimulation. Brain Stimul. 2022;15(1):141–151. 34861412 10.1016/j.brs.2021.11.015 PMC8816873

[54] Merrill DR, Bikson M, Jefferys JGR. Electrical stimulation of excitable tissue: Design of efficacious and safe protocols. J Neurosci Methods. 2005;141(2):171–198. 15661300 10.1016/j.jneumeth.2004.10.020

[55] Lilly JC, Hughes JR, Alvord EC, Galkin TW. Brief, noninjurious electric waveform for stimulation of the brain. Science. 1955;121(3144):468–469. 14358670 10.1126/science.121.3144.468

[56] Watson M, Dancause N, Sawan M. Intracortical microstimulation parameters dictate the amplitude and latency of evoked responses. Brain Stimul. 2016;9(2):276–284. 26633857 10.1016/j.brs.2015.10.008

[57] Stieger KC, Eles JR, Ludwig KA, Kozai TDY. Intracortical microstimulation pulse waveform and frequency recruits distinct spatiotemporal patterns of cortical neuron and neuropil activation. J Neural Eng. 2022;19(2): Article 026024. 10.1088/1741-2552/ac5bf5 PMC9171725 35263736

[58] Wu GK, Ardeshirpour Y, Mastracchio C, Kent J, Caiola M, Ye M. Amplitude- and frequency-dependent activation of layer II/III neurons by intracortical microstimulation. IScience. 2023;26(11): Article 108140. 37915592 10.1016/j.isci.2023.108140 PMC10616374

[59] Kumaravelu K, Grill WM. Neural mechanisms of the temporal response of cortical neurons to intracortical microstimulation. Brain Stimul. 2024;17(2):365–381. 38492885 10.1016/j.brs.2024.03.012 PMC11090107

[60] Kozai TDY, Hooks BM, Vazquez AL, Gharbawie OA, Huang C, Graczyk E, Smith MA, Taylor DM, Hernandez-Reynoso AG, London AJ, et al. Solving the problem of inception: A cross-species perspective on strategies for a mechanistic refinement of intracortical microstimulation. J Neural Eng. 2026;23(2): Article 023001. 10.1088/1741-2552/ae54cf 41855580

[61] Greenspon CM, Valle G, Shelchkova ND, Hobbs TG, Verbaarschot C, Callier T, Berger-Wolf EI, Okorokova EV, Hutchison BC, Dogruoz E, et al. Evoking stable and precise tactile sensations via multi-electrode intracortical microstimulation of the somatosensory cortex. Nat Biomed Eng. 2025;9(6):935–951. 39643730 10.1038/s41551-024-01299-z PMC12176618

[62] Valle G, Alamri AH, Downey JE, Lienkämper R, Jordan PM, Sobinov AR, Endsley LJ, Prasad D, Boninger ML, Collinger JL, et al. Tactile edges and motion via patterned microstimulation of the human somatosensory cortex. Science. 2025;387(6731):315–322. 39818881 10.1126/science.adq5978 PMC11994950

[63] Hobbs TG, Greenspon CM, Verbaarschot C, Valle G, Hughes CL, Boninger ML, Bensmaia SJ, Gaunt RA. Biomimetic stimulation patterns drive natural artificial touch percepts using intracortical microstimulation in humans. J Neural Eng. 2025;22(3): Article 036014. 10.1088/1741-2552/adc2d4 PMC13571258 40106898

[64] Verbaarschot C, Karapetyan V, Greenspon CM, Boninger ML, Bensmaia SJ, Sorger B, Gaunt RA. Conveying tactile object characteristics through customized intracortical microstimulation of the human somatosensory cortex. Nat Commun. 2025;16(1):4017. 40312384 10.1038/s41467-025-58616-6 PMC12046030

[65] Callier T, Brantly NW, Caravelli A, Bensmaia SJ. The frequency of cortical microstimulation shapes artificial touch. Proc Natl Acad Sci. 2020;117(2):1191–1200. 31879342 10.1073/pnas.1916453117 PMC6969512

[66] Fernández E, Alfaro A, Soto-Sánchez C, Gonzalez-Lopez P, Lozano AM, Peña S, Grima MD, Rodil A, Gómez B, Chen X, et al. Visual percepts evoked with an intracortical 96-channel microelectrode array inserted in human occipital cortex. J Clin Invest. 2021;131(23). 10.1172/JCI151331 PMC8631600 34665780

[67] Schmidt EM, Bak MJ, Hambrecht FT, Kufta CV, O’Rourke DK, Vallabhanath P. Feasibility of a visual prosthesis for the blind based on intracortical micro stimulation of the visual cortex. Brain. 1996;119(2):507–522. 8800945 10.1093/brain/119.2.507

[68] Fitzsimmons NA, Drake W, Hanson TL, Lebedev MA, Nicolelis M. Primate reaching cued by multichannel spatiotemporal cortical microstimulation. J Neurosci. 2007;27(21):5593–5602. 17522304 10.1523/JNEUROSCI.5297-06.2007 PMC6672750

[69] O’Doherty JE, Lebedev M, Hanson TL, Fitzsimmons N, Nicolelis MAL. A brain-machine interface instructed by direct intracortical microstimulation. Front Integr Neurosci. 2009;3:803. 10.3389/neuro.07.020.2009 PMC2741294 19750199

[70] O’Doherty JE, Lebedev MA, Ifft PJ, Zhuang KZ, Shokur S, Bleuler H, Nicolelis MA. Active tactile exploration using a brain–machine–brain interface. Nature. 2011;479(7372):228–231. 21976021 10.1038/nature10489 PMC3236080

[71] Thomson EE, Carra R, Nicolelis MAL. Perceiving invisible light through a somatosensory cortical prosthesis. Nat Commun. 2013;4(1):1482. 23403583 10.1038/ncomms2497 PMC3674834

[72] Richardson AG, Ghenbot Y, Liu X, Hao H, Rinehart C, DeLuccia S, Torres Maldonado S, Boyek G, Zhang M, Aflatouni F, et al. Learning active sensing strategies using a sensory brain-machine interface. Proc Natl Acad Sci USA. 2019;116(35):17509–17514. 31409713 10.1073/pnas.1909953116 PMC6717311

[73] Senneka SJ, Dadarlat MC. Integration of learned artificial sensation with vision during freely moving navigation. Proc Natl Acad Sci. 2026;123(9): Article e2521769123. 41758662 10.1073/pnas.2521769123 PMC12956819

[74] Dadarlat MC, O’Doherty JE, Sabes PN. A learning-based approach to artificial sensory feedback leads to optimal integration. Nat Neurosci. 2015;18(1):138–144. 25420067 10.1038/nn.3883 PMC4282864

[75] Rebesco JM, Miller LE. Enhanced detection threshold for *in vivo* cortical stimulation produced by Hebbian conditioning. J Neural Eng. 2011;8(1): Article 016011. 21252415 10.1088/1741-2560/8/1/016011 PMC3056083

[76] Rebesco JM, Stevenson IH, Koerding K, Solla SA, Miller LE. Rewiring neural interactions by micro-stimulation. Front Syst Neurosci. 2010;4:39. 20838477 10.3389/fnsys.2010.00039 PMC2936935

[77] Guggenmos DJ, Azin M, Barbay S, Mahnken JD, Dunham C, Mohseni P, Nudo RJ. Restoration of function after brain damage using a neural prosthesis. Proc Natl Acad Sci. 2013;110(52):21177–21182. 24324155 10.1073/pnas.1316885110 PMC3876197

[78] Seeman SC, Mogen BJ, Fetz EE, Perlmutter SI. Paired stimulation for spike-timing-dependent plasticity in primate sensorimotor cortex. J Neurosci. 2017;37(7):1935–1949. 28093479 10.1523/JNEUROSCI.2046-16.2017 PMC5320619

[79] McCreery D, Han M, Pikov V, Miller C. Configuring intracortical microelectrode arrays and stimulus parameters to minimize neuron loss during prolonged intracortical electrical stimulation. Brain Stimul. 2021;14(6):1553–1562. 34678487 10.1016/j.brs.2021.10.385 PMC8800486

[80] Shannon RV. A model of safe levels for electrical stimulation. IEEE Trans Biomed Eng. 1992;39(4):424–426. 1592409 10.1109/10.126616

[81] Cogan SF, Ludwig KA, Welle CG, Takmakov P. Tissue damage thresholds during therapeutic electrical stimulation. J Neural Eng. 2016;13(2): Article 021001. 26792176 10.1088/1741-2560/13/2/021001 PMC5386002

[82] Suematsu N, Vazquez AL, Kozai TDY. Chronic alteration of Ca^2+^ and hemodynamic signals induced by intracortical microstimulation in the visual cortex of awake mice. Biomaterials. 2026;334: Article 124276.42097024 10.1016/j.biomaterials.2026.124276

[83] Doty RW, Rutledge LT, Larsen RM. Conditioned reflexes established to electrical stimulation of cat cerebral cortex. J Neurophysiol. 1956;19(5):401–415. 13367871 10.1152/jn.1956.19.5.401

[84] Tariciotti L, Mattioli L, Viganò L, Gallo M, Gambaretti M, Sciortino T, Gay L, Conti Nibali M, Gallotti A, Cerri G, et al. Object-oriented hand dexterity and grasping abilities, from the animal quarters to the neurosurgical OR: A systematic review of the underlying neural correlates in non-human, human primate and recent findings in awake brain surgery. Front Integr Neurosci. 2024;18:1324581. 38425673 10.3389/fnint.2024.1324581 PMC10902498

[85] Brown AR, Mitra S, Teskey GC, Boychuk JA. Complex forelimb movements and cortical topography evoked by intracortical microstimulation in male and female mice. Cereb Cortex. 2023;33(5):1866–1875. 35511684 10.1093/cercor/bhac178 PMC9977357

[86] Gronlier E, Vendramini E, Volle J, Wozniak-Kwasniewska A, Antón Santos N, Coizet V, Duveau V, David O. Single-pulse electrical stimulation methodology in freely moving rat. J Neurosci Methods. 2021;353: Article 109092. 33549638 10.1016/j.jneumeth.2021.109092

[87] Bundy DT, Barbay S, Hudson HM, Frost SB, Nudo RJ, Guggenmos DJ. Stimulation-evoked effective connectivity (SEEC): An in-vivo approach for defining mesoscale corticocortical connectivity. J Neurosci Methods. 2023;384: Article 109767. 36493978 10.1016/j.jneumeth.2022.109767 PMC9821523

[88] Flesher SN, Downey JE, Weiss JM, Hughes CL, Herrera AJ, Tyler-Kabara EC, Boninger ML, Collinger JL, Gaunt RA. A brain-computer interface that evokes tactile sensations improves robotic arm control. Science. 2021;372(6544):831–836. 34016775 10.1126/science.abd0380 PMC8715714

[89] Armenta Salas M, Bashford L, Kellis S, Jafari M, Jo H, Kramer D, Shanfield K, Pejsa K, Lee B, Liu CY, et al. Proprioceptive and cutaneous sensations in humans elicited by intracortical microstimulation. ELife. 2018;7: Article e32904. 29633714 10.7554/eLife.32904 PMC5896877

[90] Hughes CL, Flesher SN, Weiss JM, Boninger M, Collinger JL, Gaunt RA. Perception of microstimulation frequency in human somatosensory cortex. ELife. 2021;10: Article e65128. 34313221 10.7554/eLife.65128 PMC8376245

[91] Davis TS, Parker RA, House PA, Bagley E, Wendelken S, Normann RA, Greger B. Spatial and temporal characteristics of V1 microstimulation during chronic implantation of a microelectrode array in a behaving macaque. J Neural Eng. 2012;9(6): Article 065003. 23186948 10.1088/1741-2560/9/6/065003 PMC3521049

[92] Najarpour Foroushani A, Pack CC, Sawan M. Cortical visual prostheses: From microstimulation to functional percept. J Neural Eng. 2018;15(2): Article 021005. 29350199 10.1088/1741-2552/aaa904

[93] Hughes CL, Flesher SN, Weiss JM, Downey JE, Boninger M, Collinger JL, Gaunt RA. Neural stimulation and recording performance in human sensorimotor cortex over 1500 days. J Neural Eng. 2021;18(4): Article 045012. 10.1088/1741-2552/ac18ad PMC8500669 34320481

[94] O’Doherty JE, Shokur S, Medina LE, Lebedev MA, Nicolelis MAL. Creating a neuroprosthesis for active tactile exploration of textures. Proc Natl Acad Sci. 2019;116(43):21821–21827. 31591224 10.1073/pnas.1908008116 PMC6815176

[95] Mazurek KA, Schieber MH. Injecting instructions into premotor cortex. Neuron. 2017;96(6):1282–1289.e4. 29224724 10.1016/j.neuron.2017.11.006 PMC5739962

[96] Zhang Q, Liu B, Wang Z, Zhou J, Yang X, Zhou Q, Zhao Y, Li S, Zhou J, Wang C. Training-free regulation of grasping by intracortical tactile feedback designed via S1-M1 communication. Adv Sci. 2025;12(36): Article e03011. 10.1002/advs.202503011 PMC12462993 40586134

[97] Nudo RJ, Jenkins WM, Merzenieh MM. Repetitive microstimulation alters the cortical representation of movements in adult rats. Somatosens Mot Res. 1990;7(4):463–483. 2291378 10.3109/08990229009144720

[98] Recanzone GH, Merzenich MM, Dinse HR. Expansion of the cortical representation of a specific skin field in primary somatosensory cortex by intracortical microstimulation. Cereb Cortex. 1992;2(3):181–196. 1511220 10.1093/cercor/2.3.181

[99] Teskey GC, Monfils MH, VandenBerg PM, Kleim JA. Motor map expansion following repeated cortical and limbic seizures is related to synaptic potentiation. Cereb Cortex. 2002;12(1):98–105. 11734536 10.1093/cercor/12.1.98

[100] Dadarlat MC, Canfield RA, Orsborn AL. Neural plasticity in sensorimotor brain-machine interfaces. Annu Rev Biomed Eng. 2023;8(25):51–76. 10.1146/annurev-bioeng-110220-110833 PMC10791144 36854262

[101] Espinosa JS, Stryker MP. Development and plasticity of the primary visual cortex. Neuron. 2012;75(2):230–249. 22841309 10.1016/j.neuron.2012.06.009 PMC3612584

[102] Mustafa MA. Neuroplasticity in recovery after stroke: Mechanisms and therapeutic targets. J Physiol Biol Sci. 2025;1(1):1–11.

[103] Hebb DO. The organization of behavior; a neuropsychological theory Oxford (England): Wiley; 1949.

[104] Bharmauria V, Ouelhazi A, Lussiez R, Molotchnikoff S. Adaptation-induced plasticity in the sensory cortex. J Neurophysiol. 2022;128(4):946–962. 36130163 10.1152/jn.00114.2022

[105] Brown TH, Chapman PF, Kairiss EW, Keenan CL. Long-term synaptic potentiation. Science. 1988;242(4879):724–748. 2903551 10.1126/science.2903551

[106] Dudek SM, Bear MF. Homosynaptic long-term depression in area CA1 of hippocampus and effects of N-methyl-D-aspartate receptor blockade. Proc Natl Acad Sci. 1992;89(10):4363–4367. 1350090 10.1073/pnas.89.10.4363 PMC49082

[107] Brzosko Z, Mierau SB, Paulsen O. Neuromodulation of spike-timing-dependent plasticity: Past, present, and future. Neuron. 2019;103(4):563–581. 31437453 10.1016/j.neuron.2019.05.041

[108] Harnett MT, Bernier BE, Ahn KC, Morikawa H. Burst-timing-dependent plasticity of NMDA receptor-mediated transmission in midbrain dopamine neurons. Neuron. 2009;62(6):826–838. 19555651 10.1016/j.neuron.2009.05.011 PMC2702773

[109] Paludo Silveira JA, Protachevicz PR, Viana RL, Batista AM. Effects of burst-timing-dependent plasticity on synchronous behaviour in neuronal network. Neurocomputing. 2021;436:126–135.

[110] Timm F, Kuehn E. A mechanical stimulation glove to induce Hebbian plasticity at the fingertip. Front Hum Neurosci. 2020;14:177. 32528264 10.3389/fnhum.2020.00177 PMC7263020

[111] Prosper A, Blanchard T, Lunghi C. The interplay between Hebbian and homeostatic plasticity in the adult visual cortex. J Physiol. 2025;603(6):1521–1540. 40019812 10.1113/JP287665 PMC11908499

[112] Tang H, Riley MR, Singh B, Qi XL, Blake DT, Constantinidis C. Prefrontal cortical plasticity during learning of cognitive tasks. Nat Commun. 2022;13(1):90. 35013248 10.1038/s41467-021-27695-6 PMC8748623

[113] Eles JR, Stieger KC, Kozai TDY. The temporal pattern of intracortical microstimulation pulses elicits distinct temporal and spatial recruitment of cortical neuropil and neurons. J Neural Eng. 2021;18(1): Article 015001. 10.1088/1741-2552/abc29c PMC8167825 33075762

[114] Moorjani S, McPherson JG, Perlmutter SI. Electrical conditioning for spike-timing-dependent plasticity of neural circuits. In: *Encyclopedia of Computational Neuroscience*. New York (NY): Springer; 2020. p. 1–8 [accessed 1 Mar 2026]. https://link.springer.com/rwe/10.1007/978-1-4614-7320-6_100700-1

[115] Song W, Kerr CC, Lytton WW, Francis JT. Cortical plasticity induced by spike-triggered microstimulation in primate somatosensory cortex. PLOS ONE. 2013;8(3): Article e57453. 23472086 10.1371/journal.pone.0057453 PMC3589388

[116] Andrade-Talavera Y, Fisahn A, Rodríguez-Moreno A. Timing to be precise? An overview of spike timing-dependent plasticity, brain rhythmicity, and glial cells interplay within neuronal circuits. Mol Psychiatry. 2023;28(6):2177–2188. 36991134 10.1038/s41380-023-02027-w PMC10611582

[117] Averna A, Pasquale V, Murphy MD, Rogantin MP, Van Acker GM, Nudo RJ, Chiappalone M, Guggenmos DJ. Differential effects of open- and closed-loop intracortical microstimulation on firing patterns of neurons in distant cortical areas. Cereb Cortex. 2020;30(5):2879–2896. 31832642 10.1093/cercor/bhz281 PMC7197073

[118] Averna A, Hayley P, Murphy MD, Barban F, Nguyen J, Buccelli S, Nudo RJ, Chiappalone M, Guggenmos DJ. Entrainment of network activity by closed-loop microstimulation in healthy ambulatory rats. Cereb Cortex. 2021;31(11):5042–5055. 34165137 10.1093/cercor/bhab140 PMC8491688

[119] Barban F, Averna A, Murphy MD, Carè M, Nudo RJ, Guggenmos DJ, Chiappalone M. LFP based analysis of brain injured anesthetized animals undergoing closed-loop intracortical stimulation. In: *2021 10th International IEEE/EMBS Conference on Neural Engineering (NER)*. IEEE; 2021. p. 293–296 [accessed 22 Feb 2026]. https://ieeexplore.ieee.org/document/9441266

[120] Carè M, Averna A, Barban F, Murphy MD, Nudo RJ, Guggenmos DJ, Chiappalone M. Spike-based analysis of brain injured anesthetized animals undergoing closed-loop intracortical stimulation. In: *2021 10th International IEEE/EMBS Conference on Neural Engineering (NER)*. IEEE; 2021. p. 285–288 [accessed 22 Feb 2026]. https://ieeexplore.ieee.org/document/9441303

[121] Carè M, Averna A, Barban F, Semprini M, De Michieli L, Nudo RJ, Guggenmos DJ, Chiappalone M. The impact of closed-loop intracortical stimulation on neural activity in brain-injured, anesthetized animals. Bioelectron Med. 2022;8(1):4. 35220964 10.1186/s42234-022-00086-y PMC8883660

[122] Mavoori J, Jackson A, Diorio C, Fetz E. An autonomous implantable computer for neural recording and stimulation in unrestrained primates. J Neurosci Methods. 2005;148(1):71–77. 16102841 10.1016/j.jneumeth.2005.04.017

[123] Zanos S, Richardson AG, Shupe L, Miles FP, Fetz EE. The Neurochip-2: An autonomous head-fixed computer for recording and stimulating in freely behaving monkeys. IEEE Trans Neural Syst Rehabil Eng. 2011;19(4):427–435. 21632309 10.1109/TNSRE.2011.2158007 PMC3159515

[124] Shupe LE, Miles FP, Jones G, Yun R, Mishler J, Rembado I, Murphy RL, Perlmutter SI, Fetz EE. Neurochip3: An autonomous multichannel bidirectional brain-computer Interface for closed-loop activity-dependent stimulation. Front Neurosci. 2021;15:718465. 34489634 10.3389/fnins.2021.718465 PMC8417105

[125] Potter SM. A low-cost multielectrode system for data acquisition enabling real-time closed-loop processing with rapid recovery from stimulation artifacts. Front Neuroeng. 2009;2:555. 10.3389/neuro.16.012.2009 PMC2722905 19668698

[126] Newman JP, Zeller-Townson R, Fong M, Arcot Desai S, Gross RE, Potter SM. Closed-loop, multichannel experimentation using the open-source NeuroRighter electrophysiology platform. Front Neural Circuits. 2013;6:98. 23346047 10.3389/fncir.2012.00098 PMC3548271

[127] Zhou A, Santacruz SR, Johnson BC, Alexandrov G, Moin A, Burghardt FL, Rabaey JM, Carmena JM, Muller R. A wireless and artefact-free 128-channel neuromodulation device for closed-loop stimulation and recording in non-human primates. Nat Biomed Eng. 2019;3(1):15–26. 30932068 10.1038/s41551-018-0323-x

[128] Rizzuto DS, Herrema HG, Hu Z, Utin D, Kahn J, Ho C, Smiles AB, Gross RE, Lega BC, Das SR, et al. A wireless, 60-channel, AI-enabled neurostimulation platform. Brain Stimul. 2026;19(1): Article 103013. 41429310 10.1016/j.brs.2025.103013

[129] Lee J, Leung V, Lee AH, Huang J, Asbeck P, Mercier PP, Shellhammer S, Larson L, Laiwalla F, Nurmikko A. Neural recording and stimulation using wireless networks of microimplants. Nat Electron. 2021;4(8):604–614.

[130] Lee J, Lee AH, Leung V, Laiwalla F, Lopez-Gordo MA, Larson L, Nurmikko A. An asynchronous wireless network for capturing event-driven data from large populations of autonomous sensors. Nat Electron. 2024;7(4):313–324. 38737565 10.1038/s41928-024-01134-y PMC11078753

[131] Zhu B, Shin U, Shoaran M. Closed-loop neural prostheses with on-chip intelligence: A review and a low-latency machine learning model for brain state detection. IEEE Trans Biomed Circuits Syst. 2021;15(5):877–897. 34529573 10.1109/TBCAS.2021.3112756 PMC8733782

[132] Tsakanika E, Tsoukas V, Kakarountas A, Kokkinos V. High accuracy of epileptic seizure detection using tiny machine learning technology for implantable closed-loop neurostimulation systems. BioMedInformatics. 2025;5(1):14.

[133] Galanis C, Vlachos A. Hebbian and homeostatic synaptic plasticity—Do alterations of one reflect enhancement of the other? Front Cell Neurosci. 2020;14:50. 32256317 10.3389/fncel.2020.00050 PMC7093376

[134] Bienenstock EL, Cooper LN, Munro PW. Theory for the development of neuron selectivity: Orientation specificity and binocular interaction in visual cortex. J Neurosci. 1982;2(1):32–48. 7054394 10.1523/JNEUROSCI.02-01-00032.1982 PMC6564292

[135] Abraham WC, Bear MF. Metaplasticity: The plasticity of synaptic plasticity. Trends Neurosci. 1996;19(4):126–130. 8658594 10.1016/s0166-2236(96)80018-x

[136] Turrigiano GG, Leslie KR, Desai NS, Rutherford LC, Nelson SB. Activity-dependent scaling of quantal amplitude in neocortical neurons. Nature. 1998;391(6670):892–896. 9495341 10.1038/36103

[137] Chen L, Li X, Tjia M, Thapliyal S. Homeostatic plasticity and excitation-inhibition balance: The good, the bad, and the ugly. Curr Opin Neurobiol. 2022;75: Article 102553. 35594578 10.1016/j.conb.2022.102553 PMC9477500

[138] Curia G. Hebbian and homeostatic synaptic plasticity of AMPA receptors in epileptogenesis. Cell Rep Med. 2023;4(5): Article 101047. 37196628 10.1016/j.xcrm.2023.101047 PMC10213967

[139] Schiller Y, Bankirer Y. Cellular mechanisms underlying antiepileptic effects of low- and high-frequency electrical stimulation in acute epilepsy in neocortical brain slices *in vitro*. J Neurophysiol. 2007;97(3):1887–1902. 17151229 10.1152/jn.00514.2006

[140] Chang WC, Kudlacek J, Hlinka J, Chvojka J, Hadrava M, Kumpost V, Powell AD, Janca R, Maturana MI, Karoly PJ, et al. Loss of neuronal network resilience precedes seizures and determines the ictogenic nature of interictal synaptic perturbations. Nat Neurosci. 2018;21(12):1742–1752. 30482946 10.1038/s41593-018-0278-y PMC7617160

[141] Dahal P, Ghani N, Flinker A, Dugan P, Friedman D, Doyle W, Devinsky O, Khodagholy D, Gelinas JN. Interictal epileptiform discharges shape large-scale intercortical communication. Brain. 2019;142(11):3502–3513. 31501850 10.1093/brain/awz269 PMC6821283

[142] Ferrero JJ, Hassan AR, Yu Z, Zhao Z, Ma L, Wu C, Shao S, Kawano T, Engel J, Doyle W, et al. Closed-loop electrical stimulation prevents focal epilepsy progression and long-term memory impairment. Nat Neurosci. 2025;28(8):1753–1762. 40551024 10.1038/s41593-025-01988-1 PMC12321579

[143] Lundstrom BN, Van Gompel J, Britton J, Nickels K, Wetjen N, Worrell G, Stead M. Chronic subthreshold cortical stimulation to treat focal epilepsy. JAMA Neurol. 2016;73(11):1370–1372. 27654625 10.1001/jamaneurol.2016.2857 PMC13095147

[144] Zhao Z, Cea C, Gelinas JN, Khodagholy D. Responsive manipulation of neural circuit pathology by fully implantable, front-end multiplexed embedded neuroelectronics. Proc Natl Acad Sci. 2021;118(20): Article e2022659118. 33972429 10.1073/pnas.2022659118 PMC8157942

[145] Ryvlin P, Rheims S, Hirsch LJ, Sokolov A, Jehi L. Neuromodulation in epilepsy: State-of-the-art approved therapies. Lancet Neurol. 2021;20(12):1038–1047. 34710360 10.1016/S1474-4422(21)00300-8

[146] Rebelo D, Oliveira F, Abrunhosa A, Januário C, Lemos J, Castelo-Branco M. A link between synaptic plasticity and reorganization of brain activity in Parkinson’s disease. Proc Natl Acad Sci. 2021;118(3): Article e2013962118. 33431672 10.1073/pnas.2013962118 PMC7826364

[147] Sciacca G, Mostile G, Disilvestro I, Donzuso G, Nicoletti A, Zappia M. Long-duration response to levodopa, motor learning, and neuroplasticity in early Parkinson’s disease. Mov Disord. 2023;38(4):626–635. 36840442 10.1002/mds.29344

[148] Zhuang X, Mazzoni P, Kang UJ. The role of neuroplasticity in dopaminergic therapy for Parkinson disease. Nat Rev Neurol. 2013;9(5):248–256. 23588357 10.1038/nrneurol.2013.57

[149] Rivlin-Etzion M, Marmor O, Saban G, Rosin B, Haber SN, Vaadia E, Prut Y, Bergman H. Low-pass filter properties of basal ganglia–cortical–muscle loops in the normal and MPTP primate model of parkinsonism. J Neurosci. 2008;28(3):633–649. 18199764 10.1523/JNEUROSCI.3388-07.2008 PMC6670346

[150] Viaro R, Morari M, Franchi G. Progressive motor cortex functional reorganization following 6-hydroxydopamine lesioning in rats. J Neurosci. 2011;31(12):4544–4554. 21430155 10.1523/JNEUROSCI.5394-10.2011 PMC6622898

[151] Aparicio-Juárez A, Duhne M, Lara-González E, Ávila-Cascajares F, Calderón V, Galarraga E, Bargas J. Cortical stimulation relieves parkinsonian pathological activity *in vitro*. Eur J Neurosci. 2019;49(6):834–848. 29250861 10.1111/ejn.13806

[152] Peng RC, Liu XX, Ke Y, Yung WH. Randomized cortical stimulation could ameliorate locomotive inability in parkinsonian rats: A pilot study. Biomed Phys Eng Express. 2020;6(2): Article 027002. 33438644 10.1088/2057-1976/ab756e

[153] Grippe T, Shamli-Oghli Y, Darmani G, Nankoo JF, Raies N, Sarica C, Arora T, Gunraj C, Ding MY, Rinchon C, et al. Plasticity-induced effects of theta burst transcranial ultrasound stimulation in Parkinson’s disease. Mov Disord. 2024;39(8):1364–1374. 38787806 10.1002/mds.29836

[154] Deuschl G, Schade-Brittinger C, Krack P, Volkmann J, Schäfer H, Bötzel K, Daniels C, Deutschländer A, Dillmann U, Eisner W, et al. A randomized trial of deep-brain stimulation for Parkinson’s disease. N Engl J Med. 2006;355(9):896–908. 16943402 10.1056/NEJMoa060281

[155] Beuter A, Lefaucheur JP, Modolo J. Closed-loop cortical neuromodulation in Parkinson’s disease: An alternative to deep brain stimulation? Clin Neurophysiol. 2014;125(5):874–885. 24555921 10.1016/j.clinph.2014.01.006

[156] McIntyre CC, Grill WM. Selective microstimulation of central nervous system neurons. Ann Biomed Eng. 2000;28(3):219–213. 10784087 10.1114/1.262

[157] Suematsu N, Vazquez AL, Kozai TDY. Activation and depression of neural and hemodynamic responses induced by the intracortical microstimulation and visual stimulation in the mouse visual cortex. J Neural Eng. 2024;21(2): Article 026033. 10.1088/1741-2552/ad3853 PMC11002944 38537268

[158] Chai Z, Ma C, Jin X. Cortical stimulation for treatment of neurological disorders of hyperexcitability: A role of homeostatic plasticity. Neural Regen Res. 2019;14(1):34. 30531066 10.4103/1673-5374.243696 PMC6262991

[159] Zhao ZP, Nie C, Jiang CT, Cao SH, Tian KX, Yu S, Gu JW. Modulating brain activity with invasive brain–computer interface: A narrative review. Brain Sci. 2023;13(1):134. 36672115 10.3390/brainsci13010134 PMC9856340

[160] Bartels J, Andreasen D, Ehirim P, Mao H, Seibert S, Wright EJ, Kennedy P. Neurotrophic electrode: Method of assembly and implantation into human motor speech cortex. J Neurosci Methods. 2008;174(2):168–176. 18672003 10.1016/j.jneumeth.2008.06.030 PMC2574508

[161] Gearing M, Kennedy P. Histological confirmation of myelinated neural filaments within the tip of the neurotrophic electrode after a decade of neural recordings. Front Hum Neurosci. 2020;21(14):111. 10.3389/fnhum.2020.00111 PMC7187752 32372930

[162] Purcell EK, Seymour JP, Yandamuri S, Kipke DR. *In vivo* evaluation of a neural stem cell-seeded prosthesis. J Neural Eng. 2009;6(2): Article 026005. 19287078 10.1088/1741-2560/6/2/026005 PMC2930820

[163] De Faveri S, Maggiolini E, Miele E, De Angelis F, Cesca F, Benfenati F, Fadiga L. Bio-inspired hybrid microelectrodes: A hybrid solution to improve long-term performance of chronic intracortical implants. Front Neuroeng. 2014;7:7. 24782757 10.3389/fneng.2014.00007 PMC3989589

[164] Azemi E, Gobbel GT, Cui XT. Seeding neural progenitor cells on silicon-based neural probes. J Neurosurg. 2010;113(3):673–681. 20151783 10.3171/2010.1.JNS09313

[165] Ahmed B, Binder S, Boroomand S, Strathman HJ, Hantak M, Shepherd T, Ehrenhofer A, Zhang H, Shepherd J, Solzbacher F, et al. Implementation and testing of a biohybrid transition microelectrode array for neural recording and modulation. bioRxiv. 2023 https://www.biorxiv.org/content/10.1101/2023.08.08.552362v1

[166] Sifringer L, Fratzl A, Clément BF, Chansoria P, Mönkemöller LS, Duru J, Ihle SJ, Steffens S, Beltraminelli A, Ceylan E, et al. An implantable biohybrid neural interface toward synaptic deep brain stimulation. Adv Funct Mater. 2025;35(12):2416557.

[167] Shao W, Meng W, Zuo J, Li X, Ming D. Opportunities and challenges of brain-on-a-chip interfaces. Cyborg Bionic Syst. 2025;6:0287. 40530005 10.34133/cbsystems.0287 PMC12173028

[168] Khajehnejad M, Habibollahi F, Loefer A, Paul A, Razi A, Kagan BJ. Dynamic network plasticity and sample efficiency in biological neural cultures: A comparative study with deep reinforcement learning. Cyborg Bionic Syst. 2025;6:0336. 40762022 10.34133/cbsystems.0336 PMC12320521

[169] Guo L, Zou D, Qiu W, Fei F, Chen L, Chen W, Xiong H, Li X, Wang Y, Gao M, et al. Linc-NSC affects cell differentiation, apoptosis and proliferation in mouse neural stem cells and embryonic stem cells *in vitro* and *in vivo*. Cell Mol Life Sci. 2024;81(1):182. 38615283 10.1007/s00018-024-05224-0 PMC11016521

[170] Wang Z, Zheng D, Chou SM, Kang PJ, Ye F, Zhang SC. Transcriptional code for circuit integration in the injured brain by transplanted human neurons. Cell Stem Cell. 2026;33(1):44–57.e7. 41512834 10.1016/j.stem.2025.12.008

[171] Yamashita H, Kikuchi T, Kodaka Y, Doi D, Ikeda M, Takahashi J. Transplantation of human iPS cell-derived cerebral cortical neurons promotes fine motor recovery in a female mouse model of ischemic stroke. Stem Cell Rev Rep. 2026;22(1):555–565. 41075148 10.1007/s12015-025-10981-x PMC12795914

[172] Revah O, Gore F, Kelley KW, Andersen J, Sakai N, Chen X, Li MY, Birey F, Yang X, Saw NL, et al. Maturation and circuit integration of transplanted human cortical organoids. Nature. 2022;610(7931):319–326. 36224417 10.1038/s41586-022-05277-w PMC9556304

[173] Yuan TF, Dong Y, Zhang L, Qi J, Yao C, Wang Y, Chai R, Liu Y, So KF. Neuromodulation-based stem cell therapy in brain repair: Recent advances and future perspectives. Neurosci Bull. 2021;37(5):735–745. 33871821 10.1007/s12264-021-00667-y PMC8099989

[174] Zhang K, De Maria D, Jebakumar M, Collins J, Fox KE, Sherrell PC, Gelmi A. The bionic interface: Considering the material mediated electrical stimulation of stem cells. Adv Mater. 2026;38(2): Article e12399. 41020390 10.1002/adma.202512399 PMC12783963

[175] Li Q, Kang B, Wang L, Chen T, Zhao Y, Feng S, Li R, Zhang H. Microfluidics embedded with microelectrodes for electrostimulation of neural stem cells proliferation. Chin Chem Lett. 2022;33(3):1308–1312.

[176] Kim NY, Choi YY, Kim TH, Ha JH, Kim TH, Kang T, Chung BG. Synergistic effect of electrical and biochemical stimulation on human iPSC-derived neural differentiation in a microfluidic electrode array chip. ACS Appl Mater Interfaces. 2024;16(13):15730–15740. 38527279 10.1021/acsami.3c17108

[177] Paşca AM, Sloan SA, Clarke LE, Tian Y, Makinson CD, Huber N, Kim CH, Park JY, O’rourke NA, Nguyen KD, et al. Functional cortical neurons and astrocytes from human pluripotent stem cells in 3D culture. Nat Methods. 2015;12(7):671–678. 26005811 10.1038/nmeth.3415 PMC4489980

[178] Soscia DA, Lam D, Tooker AC, Enright HA, Triplett M, Karande P, Peters SK, Sales AP, Wheeler EK, Fischer NO. A flexible 3-dimensional microelectrode array for *in vitro* brain models. Lab Chip. 2020;20(5):901–911. 31976505 10.1039/c9lc01148j

[179] Zhang H, Huang N, Bian S, Sawan M. Platinum wire-embedded culturing device for interior signal recording from lollipop-shaped neural spheroids. Cyborg Bionic Syst. 2025;6: Article 0220. 40045948 10.34133/cbsystems.0220 PMC11880574

[180] Najar-Asl M, Halvaei M, Abolhasani R, Mirsadeghi S, Simorgh S, Rahmani S, Pooyan P, Yektadoost E, Kiani S, Abolghasemi-Dehaqani MR, et al. Enhanced development of human pluripotent stem cell-derived cerebral organoids via an electrical stimulation bioreactor. Chem Eng J. 2024;1(487): Article 150368.

[181] Lv S, He E, Luo J, Liu Y, Liang W, Xu S, Zhang K, Yang Y, Wang M, Song Y, et al. Using human-induced pluripotent stem cell derived neurons on microelectrode arrays to model neurological disease: A review. Adv Sci. 2023;10(33):2301828. 10.1002/advs.202301828 PMC10667858 37863819

[182] George PM, Bliss TM, Hua T, Lee A, Oh B, Levinson A, Mehta S, Sun G, Steinberg GK. Electrical preconditioning of stem cells with a conductive polymer scaffold enhances stroke recovery. Biomaterials. 2017;1(142):31–40. 10.1016/j.biomaterials.2017.07.020 PMC5575756 28719819

[183] Li XH, Hu N, Chang ZH, Shi JX, Fan X, Chen MM, Bao SQ, Chen C, Zuo JC, Zhang XW, et al. Brain organoid maturation and implantation integration based on electrical signals input. J Adv Res. 2025;1(73):375–395. 10.1016/j.jare.2024.08.035 PMC12225903 39243942

[184] Tang-Schomer MD, Hu X, Hronik-Tupaj M, Tien LW, Whalen MJ, Omenetto FG, Kaplan DL. Film-based implants for supporting neuron–electrode integrated interfaces for the brain. Adv Funct Mater. 2014;24(13):1938–1948. 25386113 10.1002/adfm.201303196 PMC4224295

[185] Oh B, Santhanam S, Azadian M, Swaminathan V, Lee AG, McConnell KW, Levinson A, Song S, Patel JJ, Gardner EE, et al. Electrical modulation of transplanted stem cells improves functional recovery in a rodent model of stroke. Nat Commun. 2022;13(1):1366. 35292643 10.1038/s41467-022-29017-w PMC8924243

[186] Yang H, Liu H, Lou C, Han M, Sun Z, Su Y, Bian K, Zhao D, Li Y, Sang Y, et al. Flexible living artificial dura mater for efficient therapy of central nervous system injury based on neuronal differentiation and neuroprotective A2 astrocyte activation. Adv Mater. 2025;37(45): Article e11878. 40878588 10.1002/adma.202511878

[187] Wilson MN, Thunemann M, Liu X, Lu Y, Puppo F, Adams JW, Kim JH, Ramezani M, Pizzo DP, Djurovic S, et al. Multimodal monitoring of human cortical organoids implanted in mice reveal functional connection with visual cortex. Nat Commun. 2022;13(1):7945. 36572698 10.1038/s41467-022-35536-3 PMC9792589

[188] Hu N, Shi JX, Chen C, Xu HH, Chang ZH, Hu PF, Guo D, Zhang XW, Shao WW, Fan X, et al. Constructing organoid-brain-computer interfaces for neurofunctional repair after brain injury. Nat Commun. 2024;15(1):9580. 39505863 10.1038/s41467-024-53858-2 PMC11541701

[189] Lee AH, Lee J, Leung V, Larson L, Nurmikko A. Patterned electrical brain stimulation by a wireless network of implantable microdevices. Nat Commun. 2024;15(1):10093. 39572612 10.1038/s41467-024-54542-1 PMC11582589

[190] Kunigk NG, Urdaneta ME, Malone IG, Delgado F, Otto KJ. Reducing behavioral detection thresholds per electrode via synchronous, spatially-dependent intracortical microstimulation. Front Neurosci. 2022;16:876142. 35784835 10.3389/fnins.2022.876142 PMC9247280

[191] Hughes C, Kozai T. Dynamic amplitude modulation of microstimulation evokes biomimetic onset and offset transients and reduces depression of evoked calcium responses in sensory cortices. Brain Stimul. 2023;16(3):939–965. 37244370 10.1016/j.brs.2023.05.013 PMC10330928

[192] Bosking WH, Beauchamp MS, Yoshor D. Electrical stimulation of visual cortex: Relevance for the development of visual cortical prosthetics. Annu Rev Vis Sci. 2017;3:141–166. 28753382 10.1146/annurev-vision-111815-114525 PMC6916716

[193] Nakatani M, Matsumoto R, Kobayashi K, Hitomi T, Inouchi M, Matsuhashi M, Kinoshita M, Kikuchi T, Yoshida K, Kunieda T, et al. Electrical cortical stimulations modulate spike and post-spike slow-related high-frequency activities in human epileptic foci. Clin Neurophysiol. 2020;131(8):1741–1754. 32504935 10.1016/j.clinph.2020.03.042

[194] Saalmann YB, Mofakham S, Mikell CB, Djuric PM. Microscale multicircuit brain stimulation: Achieving real-time brain state control for novel applications. Curr Res Neurobiol. 2023;4: Article 100071. 36619175 10.1016/j.crneur.2022.100071 PMC9816916

[195] Li J, Zhang W, Liao Y, Qiu Y, Zhu Y, Zhang X, Wang C. Neural decoding reliability: Breakthroughs and potential of brain–computer interfaces technologies in the treatment of neurological diseases. Phys Life Rev. 2025;1(55):1–40. 10.1016/j.plrev.2025.08.007 40850267

[196] Preszler C, Stieger KC, Chen K, Zhang G, Kozai TDY. Microglia surveillance is directed toward neuron activation during sustained intracortical microstimulation. J Neural Eng. 2026;23(1): Article 016033. 10.1088/1741-2552/ae4652 PMC12947303 41698242

[197] Hendriks S, Grady C, Ramos KM, Chiong W, Fins JJ, Ford P, Goering S, Greely HT, Hutchison K, Kelly ML, et al. Ethical challenges of risk, informed consent, and posttrial responsibilities in human research with neural devices: A review. JAMA Neurol. 2019;76(12):1506–1514. 31621797 10.1001/jamaneurol.2019.3523 PMC9395156

[198] Zeng Y, Sun K, Lu E. Declaration on the ethics of brain–computer interfaces and augment intelligence. AI Ethics. 2021;1(3):209–211.

[199] Filippi M, Yasa O, Kamm RD, Raman R, Katzschmann RK. Will microfluidics enable functionally integrated biohybrid robots? Proc Natl Acad Sci. 2022;119(35): Article e2200741119. 36001689 10.1073/pnas.2200741119 PMC9436346

[200] Krishnan SR, Liu C, Bochenek MA, Bose S, Khatib N, Walters B, O’Keeffe L, Facklam A, Langer R, Anderson DG. A wireless, battery-free device enables oxygen generation and immune protection of therapeutic xenotransplants *in vivo*. Proc Natl Acad Sci. 2023;120(40): Article e2311707120. 37738292 10.1073/pnas.2311707120 PMC10556620

[201] Fumadó Navarro J, Crilly S, Chan WK, Browne S, Mason JO, Vallejo-Giraldo C, Pandit A, Lomora M. Cerebral organoids with integrated endothelial networks emulate the neurovascular unit and mitigate core necrosis. Adv Sci. 2025;12(43): Article e07256. 10.1002/advs.202507256 PMC12631921 40884248

[202] Wu Y, Mei R, Zhou Y, Qi J, Hang C, Jiang X. Elastic thermoplastic polyurethane/graphene microneedle-mesh interfaces via microfluidic patterning for electrophysiology in neural organoids. ACS Nano. 2025;19(42):36958–36968. 41105029 10.1021/acsnano.5c08827

[203] Mardinly AR, Taylor MD, Borog A, Smith KS, Kharazia V, Roseberry TK, Neel J-C, Repak EM, Gardner TJ, Hodak MJ, et al. Cell-based brain-machine interface. United States patent US 2023/0077899 A1. 2023.

[204] Bradley DC, Troyk PR, Berg JA, Bak M, Cogan S, Erickson R, Kufta C, Mascaro M, McCreery D, Schmidt EM, et al. Visuotopic mapping through a multichannel stimulating implant in primate V1. J Neurophysiol. 2005;93(3):1659–1670. 15342724 10.1152/jn.01213.2003

